# Supersymmetry in quantum optics and in spin-orbit coupled systems

**DOI:** 10.1038/srep13097

**Published:** 2015-08-19

**Authors:** Michael Tomka, Mikhail Pletyukhov, Vladimir Gritsev

**Affiliations:** 1 Department of Physics, Boston University, 590 Commonwealth Avenue, 02215 Boston, Massachusetts, USA; 2 Institute for Theory of Statistical Physics and JARA—Fundamentals of Future Information Technology, RWTH Aachen, 52056 Aachen, Germany; 3 Institute for Theoretical Physics, University of Amsterdam, Science Park 904, Postbus 94485, 1098 XH Amsterdam, The Netherlands

## Abstract

Light-matter interaction is naturally described by coupled bosonic and fermionic subsystems. This suggests that a certain Bose-Fermi duality is naturally present in the fundamental quantum mechanical description of photons interacting with atoms. We reveal submanifolds in parameter space of a basic light-matter interacting system where this duality is promoted to a supersymmetry (SUSY) which remains unbroken. We show that SUSY is robust with respect to decoherence and dissipation. In particular, the stationary density matrix at the supersymmetric lines in parameter space has a degenerate subspace. The dimension of this subspace is given by the Witten index and thus is topologically protected. As a consequence, the dissipative dynamics is constrained by a robust additional conserved quantity which translates information about an initial state into the stationary state. In addition, we demonstrate that the same SUSY structures are present in condensed matter systems with spin-orbit couplings of Rashba and Dresselhaus types, and therefore spin-orbit coupled systems at the SUSY lines should be robust with respect to various types of disorder. Our findings suggest that optical and condensed matter systems at the SUSY points can be used for quantum information technology and can open an avenue for quantum simulation of SUSY field theories.

Supersymmetry (SUSY) is one of the most beautiful and attractive concepts in physics, since it establishes a duality between bosons and fermions, cures divergency problems and resolves the mass hierarchy in quantum field theory^1^. Furthermore, in cosmology, it can serve as an explanation of the dark matter essence^2^. If SUSY exists in nature, it needs to be a broken symmetry, since in our surrounding environments there exists no phenomenon in which a boson is converted into a fermion. Therefore, in order to observe its signatures, the common opinion is that we need powerful accelerators. However, recent progress with quantum simulators using synthetic matter (like cold atoms, trapped ions or coupled cavities systems) opened a new possibility of realizing supersymmetric systems in a nowadays laboratory. Here we show that SUSY systems can be engineered in simple and fundamental models, either by means of solid state devices or by quantum optical schemes. One implementation we discuss is based on a generalized version of the Rabi model of quantum optics, while the other one is based on the two-dimensional electron gas in a magnetic filed with the Rashba and Dresselhaus spin-orbit coupling. Further, we reveal that the manifolds in parameter space, where the SUSY is unbroken, are robust with respect to dissipation and decoherence. This suggests that SUSY systems have an advantage of being used in quantum information science.

The role of spin-orbit coupling is central for a number of current developments in low-dimensional materials, for example the spin Hall effect, the anomalous Hall effect, spintronics^3^, topological insulators and superconductors^4^,^5^ and Majorana fermions^6^. Recently, synthetic gauge fields and spin-orbit coupling have also been realized in ultracold Bose and Fermi gases with Raman beams^7^,^8^,^9^,^10^,^11^,^12^,^13^,^14^. Behind all these developments stands the simple single-particle Rashba and Dresselhaus model. We found here that spin-orbit coupled systems can possess SUSY in a broad range of parameters.

In the field of quantum optics, an even more fundamental role is played by the Jaynes-Cummings and Rabi models. These models describe a system of a single bosonic mode coupled to a two-level system via dipole interaction. The understanding of the dynamics in these models led to a breakthrough in cavity-QED systems^15^ and nanophotonics. The very presence of bosons (light quanta) and fermions (two-level systems) suggests that there is a hidden SUSY in these quantum optical models.

In this paper, we reveal explicitly the presence of a supersymmetric structure in a generalized version of the Rabi model of quantum optics. Further, we show that the generalized Rabi model can be realized in a two-dimensional electron gas with Rashba and Dresselhaus spin-orbit coupling subject to a perpendicular and constant magnetic field. In the next step, the influence of this SUSY on the dissipative dynamics of the generalized Rabi model is studied. We observe that, due to the supersymmetry the dissipative dynamics, governed by the master equation in the dressed state picture, possesses an additional conserved quantity when the system is supersymmetric. Furthermore, we studied the behavior of this additional conserved quantity, if the system slightly deviates from the supersymmetric submanifold in parameter space.

## The model and its realizations

We consider one of the simplest and most fundamental models describing the interaction of a single mode bosonic field (represented by the canonical operators â, â^†^) with a single two-level system (described by the Pauli matrices 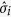, *i*  =  ±, *z*),


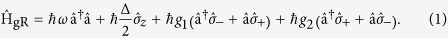


The energies of the bosonic field and the energy splitting of the two-level system are *ω* and Δ, respectively, while the interaction constants *g*_1,2_ can be arbitrary real numbers.

**Figure 1:**
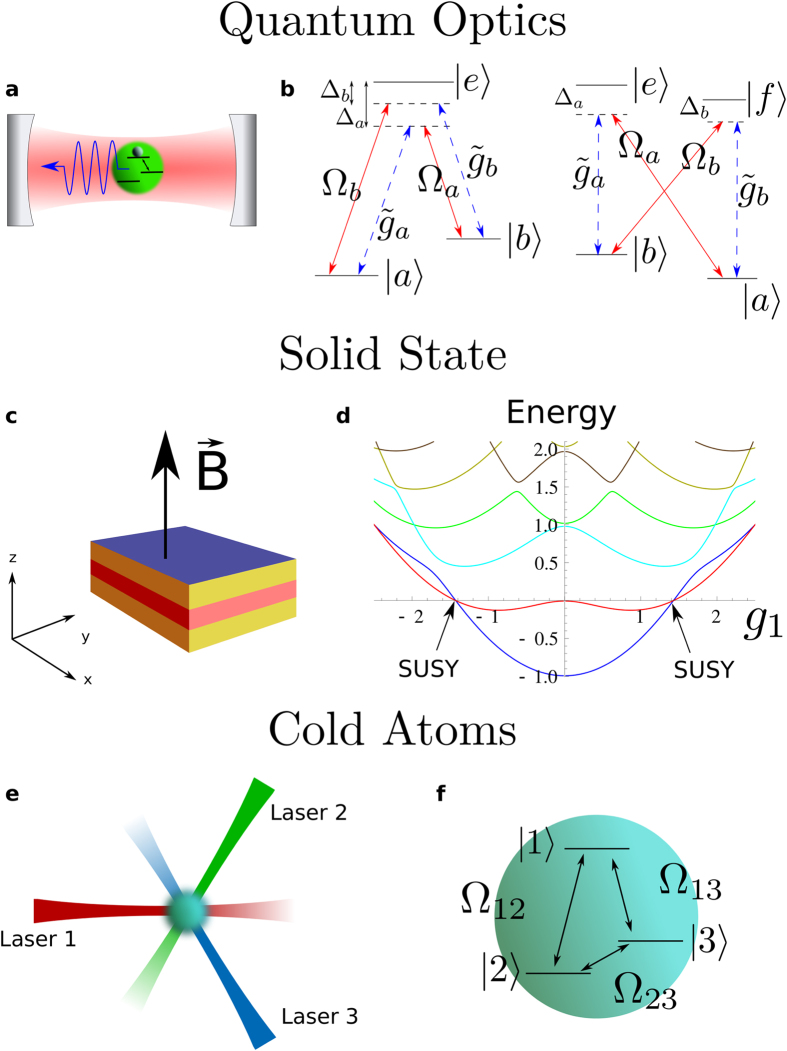
In the field of quantum optics, SUSY appears in a generalized Rabi model which can be realized in cavity-QED systems. (**a**) using the Λ-type 3- or 4-level transition schemes (**b**). In solid state systems the two-dimensional electron gas with Rashba and Dresselhaus spin-orbit couplings subject to a perpendicular magnetic field (**c**) can also be mapped to the Rabi model with unequal couplings of the co- and counter-rotating terms. In (**d**) we show the energy spectrum of these models as a function of the coupling parameter *g*_1_ ~ *α*_*R*_ and for *α*_*D*_ ~ *g*_2_ = 0.2, the SUSY lines occur when the parameters satisfy 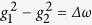, in terms of Eq. (1). In Ref. 29 a possible realization of tunable Rashba and Dresselhaus spin-orbit coupling with ultracold alkali atoms is proposed (**e**), where each state is coupled by a two-photon Raman transition, (**f**).

In the realm of quantum optics, the model (1) describes a single mode electromagnetic field interacting with a two-level emitter via dipole interaction and represents a direct generalization of two fundamental models in quantum optics. Namely, when either *g*_2_ = 0 or *g*_1_ = 0, it is known as the Jaynes-Cummings model^16^,^17^, while when *g*_1_ = *g*_2_, it becomes the Rabi model^18^,^19^. In these limits, a number of spectral and dynamical properties are known, while it is much less studied for arbitrary *g*_1_ and *g*_2_. In the weak coupling regime close to resonance *ω* ~ Δ, only the *g*_1_ term is relevant, and the *g*_2_ term scales to zero (this is called the rotating wave approximation, RWA). On the contrary, when the strong coupling regime is realized, both co- and counter-rotating terms have to be kept. We emphasize that, when the Rabi model is derived from the microscopic principles, then the coupling constants are such that *g*_1_ = *g*_2_. The Jaynes-Cummings model was studied extensively in the literature and can be solved exactly since the total number of excitations, 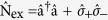, is a conserved quantity. In contrast, the analytical solution of the Rabi model is still under active discussions^20^, despite the long history of the model. Similarly to the Rabi model, the Hamiltonian (1) commutes with the parity operator 
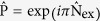
. Further, while the spectrum of the Jaynes-Cummings model is well known, the spectrum of the Rabi model is given by a self-consistent set of equations which can be solved numerically^20^. We note that in the limit of strong coupling (both *g*_1,2_/*ω* are large), the spectrum consists of two quasidegenerate harmonic ladders^21^. Both models are of immense experimental interest for circuit- and cavity-QED^15^ setups, superconducting qubits, nitrogen-vacancy (NV) centers, etc. The solid-state devices are able to approach the strong-coupling regime, where the *g*_2_ term becomes relevant^22^,^23^,^24^,^25^. In the field of quantum optics, the model with unequal *g*_1_ and *g*_2_ can be realized using the Λ-type 3- or 4-level transition schemes^26^,^27^, as shown in Fig. 1.

Indeed, consider two non-degenerate ground states |*a*〉, |*b*〉 coupled to the excited state(s), via a quantum field â, with couplings 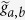. In addition, two classical laser fields with Rabi frequencies Ω_*a*,*b*_ are applied to the system, driving transitions from the ground states to the excited state(s). Δ_*a*,*b*_ denote the detunings between the lasers and the excited state(s). If 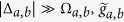 is assumed, one can perform an adiabatic elimination of the excited state(s). The resulting effective Hamiltonian then takes the form of the generalized Rabi model (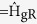) with an additional Bloch-Siegert shift, 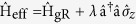 (see the Supplement for an overview of the derivation). The parameters of the generalized Rabi model are then given by 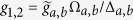, 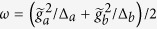, 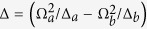 and 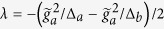. While the couplings 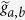 are predefined, the Rabi frequencies Ω_*a*,*b*_ as well as the detunings Δ_*a*,*b*_ can be tuned in a wide range and we can therefore consider the model (1) for variable *g*_1,2_. However, the Bloch-Seigert shift can be canceled if we choose 
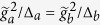
 and we obtain the generalized Rabi model (1). Ref. 28 proposes a simulation of the Rabi model with unequal *g*_1_ and *g*_2_ and with an effective Bloch-Siegert shift (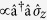) based on the resonant Raman transitions in an atom that interacts with a high finesse optical cavity mode (four-level transition scheme).

The same model appears in various branches of condensed matter science, where the spin-orbit interaction plays an important role. In particular, this is the case for a two-dimensional non-interacting electron system with Rashba and Dresselhaus spin-orbit coupling in a perpendicular magnetic field. In solid state devices, this can be realized either by the electron gas in quantum wells, in two-dimensional topological insulators or in the quantum dots with a parabolic confinement potential. In cold atomic systems, spin-orbit coupling can be achieved artificially^7^,^8^,^9^,^10^,^11^,^12^,^13^,^14^,^29^. For the case of a two-dimensional electron gas subject to a perpendicular magnetic field **B** = *B*_0_**e**_*z*_, the spin-orbit coupled Hamiltonian reads


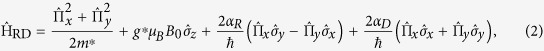


where 
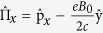
, 
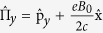
 are momentum operators in symmetric gauge, *α*_*R*_ represents the Rashba spin-orbit coupling, while *α*_*D*_ denotes the Dresselhaus spin-orbit coupling, m^*^ is the effective electron mass, g^*^ is the gyromagnetic ratio and *μ*_*B*_ = *eħ*/(2*m*_*e*_*c*) is the Bohr magneton. A short derivation of the mapping from the Hamiltonian (2) to (1) is reproduced in the Supplement. This establishes an equivalence between the electronic Rashba and Dresselhaus model with a magnetic field and the Jaynes-Cummings-Rabi model from quantum optics, which we called the generalized Rabi model.

The correspondence 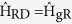 has the potential to cross-fertilize two areas of research where these models play a fundamental role: condensed matter physics and the field of quantum optics. This is illustrated below by investigating the quench dynamics in both models.

## Supersymmetry

Supersymmetric filed theories, which were studied intensively during the last 40 years, have supersymmetric quantum mechanics (SUSY QM) as their low-energy limit. Introduced in 70's the SUSY QM became a subfield by itself^30^,^31^ with many applications. Here we are interested in the *N* = 2 SUSY QM. This SUSY QM is characterized by two supercharges 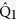 and 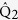 that satisfy the algebra


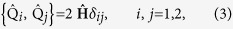


where 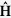 is known as the SUSY Hamiltonian acting on some Hilber space 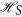. From the definition (3), it immediately follows that 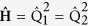. This implies that the spectrum of 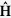 is non-negative and that the supercharges commute with the SUSY Hamiltonian, making them constants of motion. The supercharges of a SUSY QM system generate transformations between different eigenstates of the SUSY Hamiltonian with non-zero eigenenergy. This becomes more apparent when one introduces the following linear combinations of the supercharges


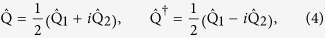


which imply 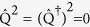 and 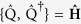. The Witten parity operator 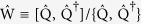 is constructed in such a way that it commutes with the SUSY Hamiltonian, anti-commutes with the supercharges and has the eigenvalues ± 1. The operator 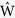 gives a natural way to decomposes the Hilbert space 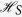 into a positive and negative Witten parity space, 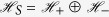, where 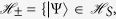 such that 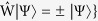. This allows us to represent the operators acting on 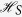 by 2 × 2 matrices. For example we can write


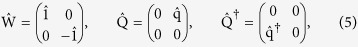


since 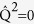 and 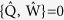. The operator 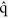 transforms a state of negative Witten parity into a state with positive Witten parity and vice versa for 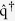. The SUSY Hamiltonian becomes diagonal in this representation


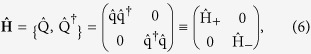


where 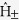 are called the super partner Hamiltonians. The supercharge 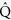 transforms a negative Witten parity eigenstate of 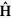 into a positive Witten parity eigenstate with the same positive energy, 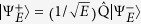 and vice versa for 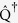. The factor 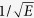 appears due to the normalization condition of the eigenstates. Therefore, the strictly positive eigenenergies of the super partner Hamiltonians 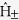 are the same.

The SUSY of a quantum system is called *unbroken* if the ground state energy of 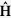 is zero (*E*_0_ = 0). In case that the ground state energy is strictly positive (*E*_0_ > 0), the SUSY is said to be *broken*. From this definition it immediately follows, that for an *unbroken* SUSY all the ground states are annihilated by all the supercharges 
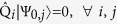
, where *j* enumerates the possible degeneracy of the ground state. This is in analogy to supersymmetric field theories, where for an *unbroken* SUSY the supercharges leave the vacuum invariant.

Our findings can be summarized as follows: 1) SUSY as a *symmetry* exists in the generalized Rabi model for a special combination of parameters,


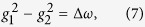


when the Bloch-Siegert shift is zero, *λ* = 0, (in the special case of *g*_1_ = *g*_2_ SUSY exists only for degenerate atomic levels, Δ = 0, and the Hamiltonian has the form of a shifted harmonic oscillator). The associated supercharges in matrix representation are given by


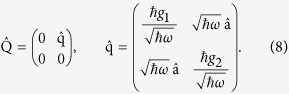


At the SUSY line (7) we can write 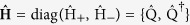, with


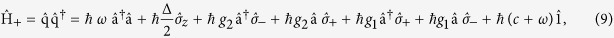



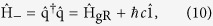


and 
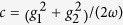
, as demonstrated in the Supplement. The generalized Rabi model is thus part of the supersymmetric system at the SUSY line (7). When *λ *≠ 0 the SUSY condition reads


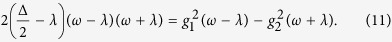


2) On the SUSY line in parameter space the SUSY is *unbroken* and only the Hamiltonian 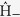 has a doubly-degenerate ground state with zero eigenenergy. The eigenenergies of the Hamiltonian 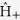 are strictly positive. Apart from the ground state of 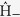 with zero eigenenergy, the Hamiltonian 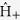 has the same spectrum as 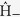 if the parameters satisfy the SUSY condition (7). This implies that the Witten index is equal two.

The Witten index *W*_ind_ is given by the difference between the number of zero eigenmodes *n* _±_ of 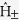, namely 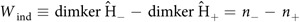. This index is related to the index of the annihilation operator 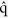, i.e. 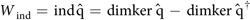, and has the property of *topological* invariance^32^ according to the Atiyah-Singer index theorem. We show explicitly in the Supplement that there are two zero eigenmodes of  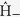, and zero for 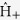, thus *W*_ind_ = 2. Similarly, to the Rabi case, the Hamiltonian (1) commutes with the parity operator 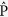, therefore the two zero-modes are the eigenstates of the parity operator 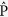 and can be written as 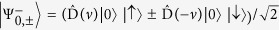, where 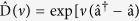 is a coherent state displacement operator with 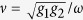 and |↑〉, |↓〉 are the eigenstates of 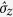. The explicit derivation of the supercharges and the zero-modes for the generalized Rabi model are given in the Supplement.

## Dissipative dynamics

In the quantum optical realization of the generalized Rabi model the effects of coupling the system to the environment are usually accounted for by the master equation in the Lindblad form. Here, we show that the SUSY in the generalized Rabi model is stable against couplings to several types of dissipative baths. Effects of relaxation and decoherence are described by the Lindblad master equation for the density matrix in the dressed picture^33^,^34^,^35^,^36^: 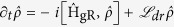, where the dissipator 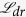 should be written in terms of the jump operators 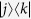 between the exact eigenstates 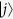, 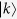 of the Hamiltonian, 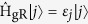,


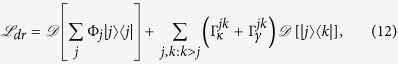


where 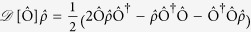 is a quantum dissipator. The different terms in Eq. (12) correspond to different sources of decoherence: The first term 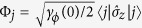, describes the diagonal part of the dephasing of the two-level system in the eigenbasis and *γ*_*ϕ*_(0) is the dephasing rate quantified by the dephasing noise spectral density at zero frequency. The other two terms describe contributions from the oscillator and the two-level system baths. They cause transitions between eigenstates with the relaxation coefficients 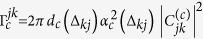, where *d*_*c*_ (Δ_*k j*_) is the spectral density of the bath and *α*_*c*_ (Δ_*k j*_) is the system-bath coupling strength at the transition frequency Δ_*kj*_ = *ε*_*k*_−*ε*_*j*_. The transition coefficients are 
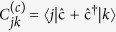
 with 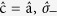. The spectral density is assumed to be constant, while 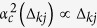. Hence, 
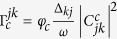
, where *φ*_*c*_ = *κ*_*c*_, *γ*_*c*_, which are the standard damping rates of a weak coupling scenario for the bosonic and spin channels of dissipation^35^.

**Figure 2:**
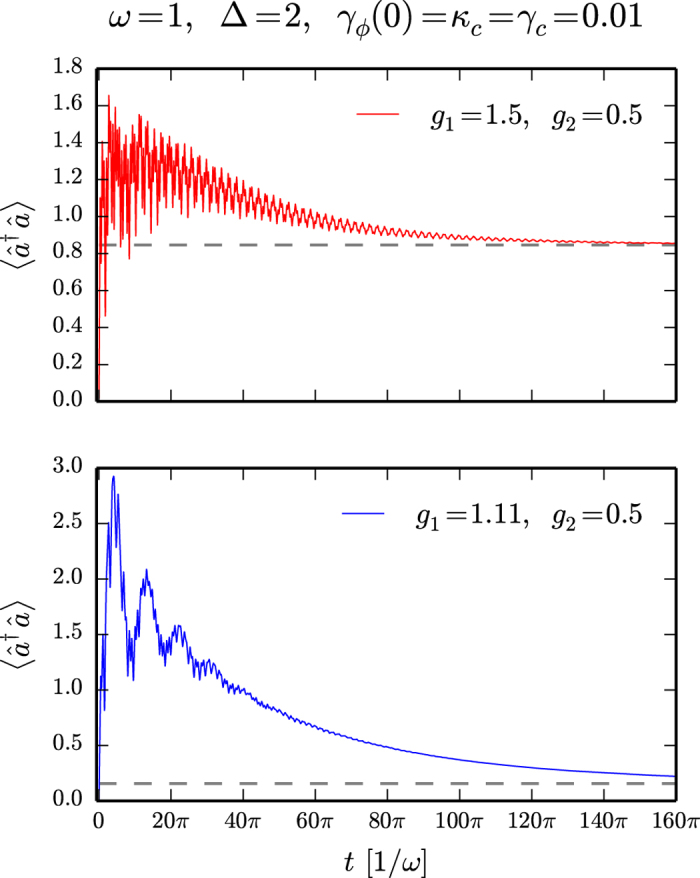
Dissipative dynamics of the generalized Rabi model. The time evolution of the mean-photon number for the initial state |0〉|↑〉 (zero photons and excited two-level system). Upper panel: evolution for parameters of the model tuned to the SUSY line (7). The stationary value (dashed line) was computed with the help of the conserved quantity *I*_1_ and *I*_2_. Lower panel: dissipation far away from the SUSY line. In this case the stationary state is given by *I*_1_, which corresponds to the trace and gives the ground state expectation value of the mean-photon number.

Using the dressed-picture dissipative formalism we checked that the dynamics preserved the trace property and that the ground state evolution has no time dependence. In Fig. 2 we illustrate the time evolution of the mean-photon number when the initial state is taken in the “spin up” state with zero bosonic occupation. The evolution at the SUSY line exhibits oscillatory behavior, while away from the SUSY line the dynamics is damped.

Usually a dissipative quantum system has a unique limit for the stationary state density matrix. However, this is not always the case. Here we found that the stationary solution of the density matrix equation has a *manifold of stationary states* at the SUSY line. Namely, the stationary solution of the Lindblad equation 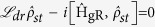, has a four-fold degenerate zero eigenvalue when *γ*_*ϕ*_(0) = 0. This manifold of the stationary states density matrices is spanned by the operators |*i*〉〈*j*|, where *i*, *j* = 1, 2 label the two degenerate states, and thus the manifold of the stationary states is equivalent to the space of unit quaternions, and can be parametrized by the SU(2) group. On the other hand, when *γ*_*ϕ*_(0) ≠ 0, only the diagonal part of this SU(2) matrix survives and the stationary state is only doubly degenerate. In the Supplement, we demonstrate that the dimension of the space of the stationary density matrices is *topologically protected* by the Witten index and the supercharge cohomology. As a consequence of the degenerate stationary subspace there is, in addition to the trace, another *conserved quantity* commuting with the Liouvillian 
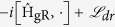
. These conserved quantities are constructed as an overlap between left and right eigenstates of the Liouvillan, 
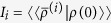
. We explicitly show how to find these conserved quantities in the Supplement. The conserved quantities can directly be used to calculate the stationary value of observables for any initial state. The conserved quantities *encode* certain information about the initial state into the stationary state. This is demonstrated in Fig. 2.

We also investigated the robustness of the SUSY-like dynamics, when we are slightly detuned from the SUSY line. We observe that the additional integral of motion, *I*_2_, becomes a time-dependent function with an extremely slow decay. Namely, for deviations up to 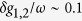 from the SUSY line, the decay can be fitted with an exponential function 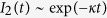 with *κ* ~ 10^−3^ for a very long time interval, corresponding to the scale of Fig. 2. This demonstrates a robustness of the SUSY-related dynamical properties even outside of the SUSY line. We attribute this behavior to the topological nature of the stationary states manifold discussed above. From a more general viewpoint this brings an analogy with the classical KAM theory, where the invariant tori stay stable for a long time.

## Cross-links: dynamics

Time evolution starting from a given initial state is very natural in the framework of quantum optics. In the Jaynes-Cummings model, when the evolution starts with a coherent state, one observes Rabi oscillations with a frequency 
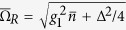
, and their collapse and revival, for a system with a large average number of photons 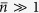
37,38. In general, three time scales can be identified: the Rabi oscillation period 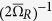, the collapse time 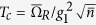 of the Rabi oscillations and the revival time 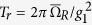, the time after which the Rabi oscillations reappear. What would be the interpretation of these phenomena in terms of the Rashba model? Consider the operator 
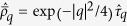
, where 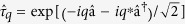 is a displacement operator. This operator is nothing but a generator of the GMP algebra of the lowest Landau Level projected density operators^39^, satisfying 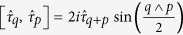, where *q*^*p* = *l*^2^(**q*** *× **p**)·**e**_*z*_ and *l *= (*ħc*/*eB*_0_)^1/2^. Therefore, by preparing the condensed matter system with Rashba spin-orbit coupling in the eigenstate |*q*〉 of the projected density operator 
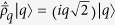
, one should be able to observe collapse and revival of the Rabi oscillations.

Still another example of cross-links between quantum optical models and spin-orbit coupled condensed matter systems could be provided by the Ramsey *π*-pulse scheme (kicks) applied to the two-level subsystem^40^,^41^. Following the previous analogy with Jaynes-Cummings model one can suggest a Ramsey spectrometry magnetic field pulse scheme to measure decoherence effects in the Rashba model.

## Coupled systems: prospects for quantum simulation of the SUSY field theories

**Figure 3:**
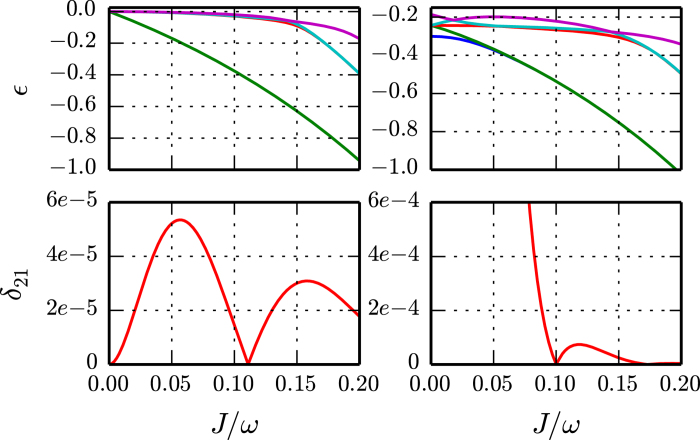
Top panels. The spectrum of a one dimensional array of 3 coupled resonators, each described by the generalized Rabi model, as a function of the tunneling amplitude *J* between the resonators, for *ω* = 1 and Δ = 2. Bottom panels: Energy difference of the lowest two levels δ_21_ = *E*_2_−*E*_1_. On the left panels the parameters are such that each generalized Rabi cavity is on the SUSY line, *g*_1_ = 1.5 and *g*_2_ = 0.5. On the right panels the parameters are chosen not to satisfy the SUSY condition, *g*_1_ = 1.4 and *g*_1_ = 0.5.

We coupled several (up to three) cavities, each described by the generalized Rabi model and tuned to the SUSY line. We observe a persistent degeneracy of the ground state in a range of the tunneling parameter, see Fig. 3. A number of recent studies suggest that coupled systems of Jaynes-Cummings- or Rabi-cavities undergo the Mott insulator-superfluid transition, and e.g., in the weak tunneling limit the coupled systems can be mapped to an effective XY-model with a magnetic field (similar to^42^). To include the effect of the tunneling between the different cavities, one should use a degenerate perturbation theory to study the SUSY points. This leads to the XY-model without an effective magnetic field. Starting from two cavities and transforming to the bonding unit-bonding basis, it is easy to show that the doubly degenerate SUSY line will exist in parameter space, although its position is altered by the tunneling rate. We conjecture that, in the continuum limit, coupled generalized Rabi cavities could be described by a continuum SUSY field theory at a specific parameter manifold. We do not exclude that the continuum model could have a critical line in parameter space, where the effective theory is a super-conformal field theory. This issue will be addressed elsewhere. Another possibility to observe SUSY, would be to design a system which is described by 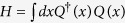, where *Q*(*x*) is a continuum analogue of 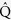 as introduced before. An experimental implementation of coupled generalized Rabi cavities, using an ensembles of NV centers coupled to superconducting microwave cavities, was recently proposed^43^.

## Discussion

Further connections between dissipative dynamics of quantum optical models and spin-orbit coupled systems can be foreseen in view of the finding of^44^: for a vanishing magnetic field and when *g*_1_ = *g*_2_, there is a SU(2) dynamical symmetry, which leads to non-diffusive spin transport in disordered spin-orbit coupled systems. The SUSY we found here has the same effect on transport for 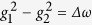 and a nonzero magnetic field Δ.

In^45^ it was found that the parity operations of a generalized Dicke model (a many-level extension of the generalized Rabi model) are discrete transformations of the electric to the magnetic field or vice versa. These transformations are best defined in terms of the electric and magnetic coupling constants Ω_*E,B*_ = *g*_1_±*g*_2_, respectively. By breaking these symmetries separately in the generalized version of the Dicke model, one can establish separate electric and magnetic phases. It is interesting to note that in this picture, our SUSY line (7) is given by Ω_*E*_Ω_*B*_ = *ω*Δ, and corresponds to the electro-magnetic self-dual line in parameter space, which is invariant under the exchange Ω_*E*_ ↔ Ω_*B*_.

We observe a nontrivial structure of the stationary state density matrix forming a SU(2) manifold. This inspires a profound study of the nontrivial topology of the density matrix encoded in dissipative dynamics and possible classification of topologically non-equivalent stationary state density matrices. An initial state density matrix is mapped to the stationary state subspace, which implies that the initial state information will be partially stored in the compact space of the stationary state manifold. This concept could be very useful for the realization of (partial) decoherence-free algorithms in the quantum information science.

## Additional Information

**How to cite this article**: Tomka, M. *et al*. Supersymmetry in quantum optics and in spin-orbit coupled systems. *Sci. Rep*. **5**, 13097; doi: 10.1038/srep13097 (2015).

## Supplementary Material

Supplementary Information
