# Supplementary material for: Supersymmetry in quantum optics and in spin-orbit coupled systems
Source: Sci Rep. 2015 Aug 19;5:13097. doi: 10.1038/srep13097 (PMC4541257; doi:10.1038/srep13097)
Supplement: Supplementary Information [file srep13097-s1.pdf]

Supplementary Material for the paper "Supersymmetry in quantum optics and in spin-orbit coupled systems" by M. Tomka, M. Pletyukhov and V. Gritsev

S1. POSSIBLE PHYSICAL REALIZATIONS OF THE GENERALIZED RABI MODEL

In this section we overview several physical realizations of the generalized Rabi model. These examples include: (i) the model of a spin-orbit interacting two-dimensional electron gas in an external magnetic field; (ii) the electric-magnetic coupling of light and matter and (iii) the effective realizations of the generalized Rabi model using 3- and 4-level emitters.

A. Rashba-Dresselhaus model in a magnetic field

For a perpendicular magnetic field $\mathbf{B} = B_0 \mathbf{e}_z$ the Hamiltonian of a two-dimensional electron gas with Rashba and Dresselhaus spin-orbit couplings is

$$\hat{\mathrm{H}}_{\mathrm{RD}} = \frac{\hat{\Pi}_x^2 + \hat{\Pi}_y^2}{2m^*} + g^* \mu_B B_0 \hat{\sigma}_z + \frac{2\alpha_R}{\hbar}(\hat{\Pi}_x \hat{\sigma}_y - \hat{\Pi}_y \hat{\sigma}_x) + \frac{2\alpha_D}{\hbar}(\hat{\Pi}_x \hat{\sigma}_x + \hat{\Pi}_y \hat{\sigma}_y), \tag{S1}$$

here $\hat{\Pi}_x = \hat{\mathrm{p}}_x - \frac{eB_0}{2c}\hat{\mathrm{y}}$, $\hat{\Pi}_y = \hat{\mathrm{p}}_y + \frac{eB_0}{2c}\hat{\mathrm{x}}$ are the in-plane momentum operators in symmetric gauge, α_R is the Rashba and α_D is the Dresselhaus spin-orbit coupling strength, m^* is the effective mass, g^* is the gyromagnetic ratio and $\mu_B = e\hbar/(2m_e c)$ is the Bohr magneton.

Due to the commutation relation between the momentum operators

$$[\hat{\Pi}_x, \hat{\Pi}_y] = i\frac{e\hbar}{c}B_0, \tag{S2}$$

one can introduce the canonically conjugated operators

$$\hat{\mathrm{Q}} = \sqrt{\frac{c}{eB_0}}\hat{\Pi}_y, \qquad \hat{\mathrm{P}} = \sqrt{\frac{c}{eB_0}}\hat{\Pi}_x, \tag{S3}$$

satisfying $[\hat{\mathrm{Q}}, \hat{\mathrm{P}}] = i\hbar$. Further, introducing the standard ladder operators

$$\hat{\mathrm{a}} = \frac{1}{\sqrt{2\hbar}}(\hat{\mathrm{Q}} + i\hat{\mathrm{P}}), \qquad \hat{\mathrm{a}}^\dagger = \frac{1}{\sqrt{2\hbar}}(\hat{\mathrm{Q}} - i\hat{\mathrm{P}}), \tag{S4}$$

such that $[\hat{\mathrm{a}}, \hat{\mathrm{a}}^\dagger] = \hat{1}$ and making use of the U(1) gauge (canonical) transformation $\hat{\mathrm{a}} \to \hat{\mathrm{a}}\, e^{i\pi/4}$, $\hat{\sigma}_+ \to \hat{\sigma}_+ \, e^{-i\pi/4}$, we obtain a Hamiltonian that is a combination of the Jaynes-Cummings and the Rabi model, namely having rotating and counter-rotating terms with different coupling strength

$$\hat{\mathrm{H}}_{\mathrm{RD}} = \hbar\omega \hat{\mathrm{a}}^\dagger \hat{\mathrm{a}} + \hbar\frac{\Delta}{2}\hat{\sigma}_z + \hbar g_1(\hat{\mathrm{a}}^\dagger \hat{\sigma}_- + \hat{\mathrm{a}}\hat{\sigma}_+) + \hbar g_2(\hat{\mathrm{a}}^\dagger \hat{\sigma}_+ + \hat{\mathrm{a}}\hat{\sigma}_-), \tag{S5}$$

where

$$\omega \equiv \frac{eB_0}{m^* c}, \qquad \Delta \equiv g^* \frac{eB_0}{m_e c}, \qquad g_{1,2} \equiv \frac{\sqrt{2}}{\hbar}\sqrt{\frac{eB_0}{\hbar c}}\frac{2\alpha_{R,D}}{\hbar}. \tag{S6}$$

B. Dipole-magnetic coupling

A complete description of the coupling between the electromagnetic field and a two-level system includes the electric dipole and magnetic dipole couplings. Namely, we consider a Hamiltonian that describes the full system

$$\hat{\mathrm{H}} = \hat{\mathrm{H}}_{\mathrm{atom}} + \hat{\mathrm{H}}_{\mathrm{field}} + \hat{\mathrm{H}}_{\mathrm{int}}, \tag{S7}$$

which consists of the atomic excitation Hamiltonian

$$\hat{\mathrm{H}}_{\mathrm{atom}} = \hbar\frac{\Delta}{2}\sigma^z, \tag{S8}$$

the free field Hamiltonian

$$\hat{\mathrm{H}}_{\mathrm{field}} = \frac{1}{2}(\mathbf{E}^2 + \mathbf{B}^2), \tag{S9}$$

and the electric and magnetic dipole interaction Hamiltonian

$$\hat{\mathrm{H}}_{\mathrm{int}} = \hat{\mathbf{d}} \cdot \mathbf{E} + \hat{\mu} \cdot \mathbf{B}. \tag{S10}$$

In the second quantized picture, the electric and the magnetic fields are given by $E \sim (a + a^\dagger)$ and $B \sim i(a - a^\dagger)$, respectively. Moreover, the dipole operators can be expressed in terms of the Pauli matrices as

$$\hat{\mathbf{d}} = \Omega_E \hat{\sigma}_x, \qquad \hat{\mu} = \Omega_B \hat{\sigma}_y, \tag{S11}$$

where $\Omega_E = g_1 + g_2$ and $\Omega_B = g_1 - g_2$, such that the Hamiltonian (S7) can be mapped to the generalized Rabi model. It was showed in Ref. [S1] that the conserved parity symmetry of the generalized Dicke model is a composite action of

$$P_E: \qquad E \to -E, \qquad \hat{d} \to -\hat{d}; \qquad B \to B, \qquad \hat{\mu} \to \hat{\mu}, \tag{S12}$$

$$P_B: \qquad B \to -B, \qquad \hat{\mu} \to -\hat{\mu}; \qquad E \to E, \qquad \hat{d} \to \hat{d}. \tag{S13}$$

We note that the SUSY line $g_1^2 - g_2^2 = \Delta\omega \Leftrightarrow \Omega_E \Omega_B = \Delta\omega$, is invariant under electric-magnetic transformation $d \leftrightarrow \mu$.

C. Effective three-level scheme

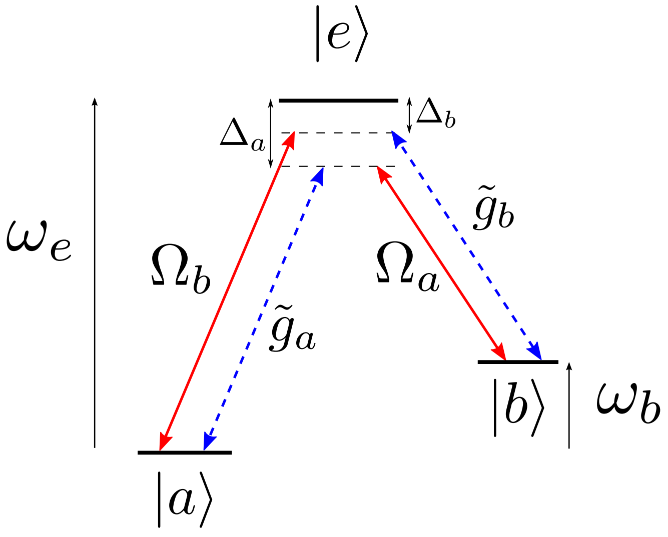

FIG. S1: Λ-type three level transition scheme ($\hbar = 1$). The energy of the ground state $|a\rangle$ is set to zero and the other ground state $|b\rangle$ has an energy ω_b, while for the excited state $|e\rangle$ we have ω_e. The quantized cavity mode couples to both transitions $|a\rangle \leftrightarrow |e\rangle$ and $|b\rangle \leftrightarrow |e\rangle$ with the rates $\tilde{g}_a$ and $\tilde{g}_b$ and with detunings Δ_a and Δ_b. In addition, two classical laser fields with Rabi frequencies Ω_a and Ω_b drive the ground state to excited state transitions with the detunings Δ_a and Δ_b, respectively.

We consider a three-level Λ-system, where the states are coupled to the quantized field of an optical cavity mode and to the classical field of a pair of lasers [S2]. The ground states are denoted by, $|a\rangle$ and $|b\rangle$, and the excited state by $|e\rangle$. The transitions $|a\rangle \leftrightarrow |e\rangle$ and $|b\rangle \leftrightarrow |e\rangle$ are both coupled to the same cavity mode with the dipole coupling strengths $\tilde{g}_a$ and $\tilde{g}_b$, respectively. The two classical laser fields with Rabi frequency Ω_b and Ω_a drive the transitions

$|a\rangle \leftrightarrow |e\rangle$ and $|b\rangle \leftrightarrow |e\rangle$. The detunings from the excited state are denoted by Δ_a and Δ_b (see Fig. S1). Transitions between the ground states levels $|a\rangle$ and $|b\rangle$ are not allowed. Therefore, the Hamiltonian, in units $\hbar = 1$, of this Λ-configuration reads

$$\begin{aligned}\hat{\mathrm{H}}_\Lambda &= \omega_{\mathrm{cav}}\, \hat{\mathrm{a}}^\dagger \hat{\mathrm{a}} + \omega_b\, |b\rangle\langle b| + \omega_e\, |e\rangle\langle e| + \\ &+ (\tilde{g}_a\, \hat{\mathrm{a}}\, |e\rangle\langle a| + \tilde{g}_b\, \hat{\mathrm{a}}\, |e\rangle\langle b| + \text{h.c.}) + \left(\Omega_b\, e^{-i\omega_{la} t}\, |e\rangle\langle a| + \Omega_a\, e^{-i\omega_{lb} t}\, |e\rangle\langle b| + \text{h.c.}\right),\end{aligned} \tag{S14}$$

where ω_b and ω_e are the energies of the ground state and the excited state (see Fig. S1), ω_{la} and ω_{lb} are the frequencies of the classical lasers and ω_{cav} is the frequency of the quantized cavity mode.

From the Hamiltonian (S14) we can derive an effective Hamiltonian that has the form of the generalized Rabi model by first going into a rotating frame and then adiabatically eliminating the excited level $|e\rangle$. First, we apply the following unitary transformation $\hat{\mathrm{R}}(t) = \exp(-i\hat{\mathrm{H}}_0 t)$, with

$$\hat{\mathrm{H}}_0 = (\omega_{lb} - \omega_b')\hat{\mathrm{a}}^\dagger \hat{\mathrm{a}} + \omega_b'\, |b\rangle\langle b| + \omega_e\, |e\rangle\langle e|, \tag{S15}$$

where ω_b' is a frequency close to ω_b given by

$$\omega_b' \equiv \frac{1}{2}(\omega_{la} - \omega_{lb}). \tag{S16}$$

The Hamiltonian in the rotating frame, $\hat{\mathrm{H}}_{\Lambda\mathrm{R}}(t) = \hat{\mathrm{R}}^\dagger(t)\hat{\mathrm{H}}_\Lambda \hat{\mathrm{R}}(t) - \hat{\mathrm{H}}_0$, reads

$$\begin{aligned}\hat{\mathrm{H}}_{\Lambda\mathrm{R}}(t) &= \delta_{\mathrm{cav}}\, \hat{\mathrm{a}}^\dagger \hat{\mathrm{a}} + (\omega_b - \omega_b')|b\rangle\langle b| + \\ &+ \left(\tilde{g}_a\, e^{i\Delta_a t}\, \hat{\mathrm{a}}\, |e\rangle\langle a| + \tilde{g}_b\, e^{i\Delta_b t}\, \hat{\mathrm{a}}\, |e\rangle\langle b| + \text{h.c.}\right) + \left(\Omega_b\, e^{i\Delta_b t}\, |e\rangle\langle a| + \Omega_a\, e^{i\Delta_a t}\, |e\rangle\langle b| + \text{h.c.}\right),\end{aligned} \tag{S17}$$

where

$$\delta_{\mathrm{cav}} \equiv \omega_{\mathrm{cav}} - (\omega_{lb} - \omega_b'), \qquad \Delta_a \equiv \omega_e - (\omega_{lb} + \omega_b'), \qquad \Delta_b \equiv \omega_e - \omega_{la}. \tag{S18}$$

Let us shorten the notation by introducing

$$\hat{\mathrm{h}}_0 \equiv \delta_{\mathrm{cav}}\, \hat{\mathrm{a}}^\dagger \hat{\mathrm{a}} + (\omega_b - \omega_b')|b\rangle\langle b|, \qquad \hat{\mathrm{A}}_a \equiv \tilde{g}_a\, \hat{\mathrm{a}}\, |e\rangle\langle a| + \Omega_a\, |e\rangle\langle b|, \qquad \hat{\mathrm{A}}_b = \tilde{g}_b\, \hat{\mathrm{a}}\, |e\rangle\langle b| + \Omega_b\, |e\rangle\langle a|, \tag{S19}$$

allowing us to rewrite (S17) as

$$\hat{\mathrm{H}}_{\Lambda\mathrm{R}}(t) = \hat{\mathrm{h}}_0 + \sum_{j=a,b} \hat{\mathrm{A}}_j e^{i\Delta_j t} + \sum_{j=a,b} \hat{\mathrm{A}}_j^\dagger e^{-i\Delta_j t}. \tag{S20}$$

The Magnus expansion [S3] allows us to derive an effective time-independent Hamiltonian from $\hat{\mathrm{H}}_{\Lambda\mathrm{R}}(t)$ by expanding the time-ordered integral of the evolution operator $\hat{\mathrm{U}}(t) = \mathcal{T} e^{-i\int_0^t dt' \hat{\mathrm{H}}_{\Lambda\mathrm{R}}(t')}$ as a series of nested commutators,

$$\hat{\mathrm{H}}_{\mathrm{eff}} = \frac{1}{t}\int_0^t dt'\, \hat{\mathrm{H}}_{\Lambda\mathrm{R}}(t') + \frac{(-i)}{2t}\int_0^t dt' \int_0^{t'} dt''\, [\hat{\mathrm{H}}_{\Lambda\mathrm{R}}(t'), \hat{\mathrm{H}}_{\Lambda\mathrm{R}}(t'')] + \dots . \tag{S21}$$

We obtain

$$\hat{\mathrm{H}}_{\mathrm{eff}} = \hat{\mathrm{h}}_0 + \sum_{j=a,b} \frac{1}{\Delta_j}\, [\hat{\mathrm{A}}_j^\dagger, \hat{\mathrm{A}}_j], \tag{S22}$$

where the other terms average to zero, if we assume large detunings of the fields from the excited state

$$|\Delta_{a,b}| \gg \Omega_{a,b}, \tilde{g}_{a,b}. \tag{S23}$$

Under these assumptions, the effective Hamiltonian (S22) is decoupled from the excited state $|e\rangle$ and we find

$$\hat{\mathrm{H}}_{\mathrm{eff}} = \omega \hat{\mathrm{a}}^\dagger \hat{\mathrm{a}} + \frac{\Delta}{2}\hat{\sigma}_z + \lambda \hat{\mathrm{a}}^\dagger \hat{\mathrm{a}} \hat{\sigma}_z + g_1(\hat{\mathrm{a}}^\dagger\, \hat{\sigma}_- + \hat{\mathrm{a}}\hat{\sigma}_+) + g_2(\hat{\mathrm{a}}^\dagger\, \hat{\sigma}_+ + \hat{\mathrm{a}}\hat{\sigma}_-), \tag{S24}$$

where the spin 1/2 operators are introduced by

$$\hat{\sigma}_z \equiv |b\rangle\langle b| - |a\rangle\langle a|, \qquad \hat{\sigma}_+ \equiv |b\rangle\langle a|, \qquad \hat{\sigma}_- \equiv |a\rangle\langle b|, \tag{S25}$$

and the parameters are defined by

$$\omega \equiv \frac{1}{2}\left(\frac{\tilde{g}_a}{\Delta_a}+\frac{\tilde{g}_b}{\Delta_b}\right)+\delta_{\rm cav}, \quad \Delta \equiv \left(\frac{\Omega_a^2}{\Delta_a}-\frac{\Omega_b^2}{\Delta_b}\right)+\frac{1}{2}(\omega_b-\omega_b'), \quad \lambda \equiv -\frac{1}{2}\left(\frac{\tilde{g}_a^2}{\Delta_a}-\frac{\tilde{g}_b^2}{\Delta_b}\right) \quad g_{1,2} \equiv \frac{\Omega_{a,b}\tilde{g}_{a,b}}{\Delta_{a,b}}. \tag{S26}$$

We neglected the constant energy terms. If however we impose the condition $\frac{\tilde{g}_a^2}{\Delta_a}=\frac{\tilde{g}_b^2}{\Delta_b}$, we get the generalized Rabi model

$$\hat{\rm H}_{\rm eff} = \omega \hat{\rm a}^\dagger \hat{\rm a} + \frac{\Delta}{2}\hat{\sigma}_z + g_1(\hat{\rm a}^\dagger\,\hat{\sigma}_- + \hat{\rm a}\hat{\sigma}_+) + g_2(\hat{\rm a}^\dagger\,\hat{\sigma}_+ + \hat{\rm a}\hat{\sigma}_-). \tag{S27}$$

Similar considerations applied to a four level scheme lead to the same effective model [S4].

S2. MATRIX REPRESENTATION OF SUPERCHARGES

The following matrix representation for the supercharge $\hat{Q}_1$ of the Jaynes-Cummings model has been suggested in [S5]

$$\hat{Q}_1 = \begin{pmatrix} 0 & 0 & \alpha & \gamma\hat{a} \\ 0 & 0 & \beta\hat{a}^\dagger & \delta \\ \alpha^* & \beta^*\hat{a} & 0 & 0 \\ \gamma^*\hat{a}^\dagger & \delta^* & 0 & 0 \end{pmatrix} = \begin{pmatrix} 0 & \hat{q} \\ \hat{q}^\dagger & 0 \end{pmatrix}. \tag{S28}$$

In [S5] they found that the Jaynes-Cummings Hamiltonian at *zero* detuning, $\omega = \frac{\Delta}{2}$, is part of the following supersymmetric Hamiltonian

$$\hat{\mathbf{H}} = \begin{pmatrix} \hat{H}_+ & 0 \\ 0 & \hat{H}_- \end{pmatrix}, \tag{S29}$$

where the two super partner Hamiltonians, in units $\hbar = 1$, are

$$\hat{H}_+ = \omega(\hat{a}^\dagger\hat{a} + \hat{\sigma}_z) + g(\hat{a}^\dagger\hat{\sigma}_- + \hat{a}\hat{\sigma}_+) + \left(\frac{1}{2}\omega + \frac{g^2}{4\omega}\right)\hat{1}, \tag{S30}$$

$$\hat{H}_- = \omega(\hat{a}^\dagger\hat{a} + \hat{\sigma}_z) + ig(\hat{a}^\dagger\hat{\sigma}_- - \hat{a}\hat{\sigma}_+) + \left(\frac{1}{2}\omega + \frac{g^2}{4\omega}\right)\hat{1}. \tag{S31}$$

If the parameters in (S28) are given by

$$\alpha = \frac{g}{2\sqrt{\omega}}, \qquad \beta = \sqrt{\omega}, \qquad \gamma = -i\sqrt{\omega}, \qquad \delta = -i\frac{g}{2\sqrt{\omega}}, \tag{S32}$$

then we have $\hat{\mathbf{H}} = \hat{Q}_1^2$, or written separately $\hat{H}_+ = \hat{q}\hat{q}^\dagger$ and $\hat{H}_- = \hat{q}^\dagger\hat{q}$.

Let us now consider possible extensions of this observation. For this purpose, we make the following ansatz for the supercharge

$$\hat{Q}_1 = \begin{pmatrix} 0 & 0 & \alpha & \gamma_1\hat{a} + \gamma_2\hat{a}^\dagger \\ 0 & 0 & \beta_1\hat{a}^\dagger + \beta_2\hat{a} & \delta \\ \alpha^* & \beta_1^*\hat{a} + \beta_2^*\hat{a}^\dagger & 0 & 0 \\ \gamma_1^*\hat{a}^\dagger + \gamma_2^*\hat{a} & \delta^* & 0 & 0 \end{pmatrix} = \begin{pmatrix} 0 & \hat{q} \\ \hat{q}^\dagger & 0 \end{pmatrix} \tag{S33}$$

and hence its square reads

$$\hat{Q}_1^2 = \begin{pmatrix} |\alpha|^2 + \hat{C}_1 & \hat{G}_1 & 0 & 0 \\ \hat{G}_1^\dagger & \hat{C}_2 + |\delta|^2 & 0 & 0 \\ 0 & 0 & |\alpha|^2 + \hat{C}_2 & \hat{G}_2 \\ 0 & 0 & \hat{G}_2^\dagger & \hat{C}_1 + |\delta|^2 \end{pmatrix} = \begin{pmatrix} \hat{q}\hat{q}^\dagger & 0 \\ 0 & \hat{q}^\dagger\hat{q} \end{pmatrix}, \tag{S34}$$

where we introduced

$$\hat{G}_1 = \alpha(\beta_1^*\hat{a} + \beta_2^*\hat{a}^\dagger) + \delta^*(\gamma_1\hat{a} + \gamma_2\hat{a}^\dagger), \tag{S35}$$

$$\hat{G}_2 = \delta(\beta_1^*\hat{a} + \beta_2^*\hat{a}^\dagger) + \alpha^*(\gamma_1\hat{a} + \gamma_2\hat{a}^\dagger), \tag{S36}$$

$$\hat{C}_1 = (\gamma_1\hat{a} + \gamma_2\hat{a}^\dagger)(\gamma_1^*\hat{a}^\dagger + \gamma_2^*\hat{a}), \tag{S37}$$

$$\hat{C}_2 = (\beta_1\hat{a} + \beta_2\hat{a}^\dagger)(\beta_1^*\hat{a} + \beta_2^*\hat{a}^\dagger). \tag{S38}$$

We would like to have $\hat{C}_1$ and $\hat{C}_2$ diagonal in the bosonic operators $\hat{a}$, $\hat{a}^\dagger$, in order to recover models like the generalized Rabi model, which is discussed in the main text. Therefore, we apply the following Bogoliubov transformation

$$\hat{a} = \mu\hat{A} + \nu\hat{A}^\dagger, \qquad \hat{a}^\dagger = \mu^* A^\dagger + \nu^*\hat{A}, \tag{S39}$$

where $|\mu|^2 - |\nu|^2 = 1$. The transformation applied to $\hat{C}_1$ yields

$$\begin{aligned} \hat{C}_1 = {} & (\hat{A}^\dagger)^2 \left(\gamma_1^*\gamma_2(\mu^*)^2 + \gamma_1\gamma_2^*\nu^2 + |\gamma_1|^2\nu\mu^* + |\gamma_2|^2\mu^*\nu\right) + \\ + {} & (\hat{A})^2 \left(\gamma_1^*\gamma_2(\nu^*)^2 + \gamma_1\gamma_2^*\mu^2 + |\gamma_1|^2\mu\nu^* + |\gamma_2|^2\nu^*\mu\right) + \\ + {} & \hat{A}^\dagger\hat{A} \left(2\gamma_1^*\gamma_2\mu^*\nu^* + 2\gamma_1\gamma_2^*\mu\nu + (|\gamma_1|^2 + |\gamma_2|^2)(|\nu|^2 + |\mu|^2)\right) + \\ + {} & \hat{1} \left(\gamma_1^*\gamma_2\mu^*\nu^* + \gamma_1\gamma_2^*\mu\nu + |\gamma_1|^2|\mu|^2 + |\gamma_2|^2|\nu|^2\right), \end{aligned} \tag{S40}$$

whereas the transformed expression of $\hat{\mathrm{C}}_2$ is obtained by replacing all $\gamma_{1,2}$ with $\beta^*_{1,2}$ in (S40). By applying the Bogoliubov transformation to $\hat{\mathrm{G}}_1$ we find

$$\begin{aligned}\hat{\mathrm{G}}_1 &= \hat{\mathrm{A}}\left(\alpha(\beta_1^*\mu+\beta_2^*\nu^*)+\delta^*(\gamma_1\mu+\gamma_2\nu^*)\right)+\\&+\hat{\mathrm{A}}^\dagger\left(\alpha(\beta_1^*\nu+\beta_2^*\mu^*)+\delta^*(\gamma_1\nu+\gamma_2\mu^*)\right),\end{aligned}\tag{S41}$$

to obtain the expression for $\hat{\mathrm{G}}_2$ we have to replace α by δ and δ by α in (S41).

Our goal is to express models like the generalized Rabi model through $\hat{\mathbf{H}}=\hat{\mathrm{Q}}_1^2$ (or written within each subspace $\hat{\mathrm{H}}_+=\hat{\mathrm{q}}\hat{\mathrm{q}}^\dagger$ and $\hat{\mathrm{H}}_-=\hat{\mathrm{q}}^\dagger\hat{\mathrm{q}}$). Therefore, we require the terms $\hat{\mathrm{A}}^2$ and $(\hat{\mathrm{A}}^\dagger)^2$ to vanish in the expression of $\hat{\mathrm{C}}_{1,2}$, which leads to the following conditions on the parameters μ,ν, $\beta_{1,2}$ and $\gamma_{1,2}$

$$(\gamma_1\nu+\gamma_2\mu^*)(\gamma_2^*\nu+\gamma_1^*\mu^*)=0,\tag{S42}$$
$$(\gamma_1\mu+\gamma_2\nu^*)(\gamma_2^*\mu+\gamma_1^*\nu^*)=0,\tag{S43}$$
$$(\beta_1^*\nu+\beta_2^*\mu^*)(\beta_2\nu+\beta_1\mu^*)=0,\tag{S44}$$
$$(\beta_1^*\mu+\beta_2^*\nu^*)(\beta_2\mu+\beta_1\nu^*)=0.\tag{S45}$$
(S46)

Further, we would like to have both, $\hat{\mathrm{A}}$ and $\hat{\mathrm{A}}^\dagger$, in the off-diagonal of $\hat{\mathrm{Q}}_1^2$ (i.e., in the $\hat{\mathrm{G}}_{1,2}$ terms), this imposes the conditions

$$\alpha(\beta_1^*\mu+\beta_2^*\nu^*)+\delta^*(\gamma_1\mu+\gamma_2\nu^*)\neq 0,\tag{S47}$$
$$\alpha(\beta_1^*\nu+\beta_2^*\mu^*)+\delta^*(\gamma_1\nu+\gamma_2\mu^*)\neq 0,\tag{S48}$$
$$\alpha^*(\gamma_1\mu+\gamma_2\nu^*)+\delta(\beta_1^*\mu+\beta_2^*\nu^*)\neq 0,\tag{S49}$$
$$\alpha^*(\gamma_1\nu+\gamma_2\mu^*)+\delta(\beta_1^*\nu+\beta_2^*\mu^*)\neq 0.\tag{S50}$$

The coefficient in front of $\hat{\mathrm{A}}^\dagger\hat{\mathrm{A}}$ in the expression of $\hat{\mathrm{C}}_1$ and $\hat{\mathrm{C}}_2$ must be the same. This gives us the last condition

$$\left(2\gamma_1^*\gamma_2\mu^*\nu^*+2\gamma_1\gamma_2^*\mu\nu+(|\gamma_1|^2+|\gamma_2|^2)(|\nu|^2+|\mu|^2)\right)\tag{S51}$$
$$=\left(2\beta_1\beta_2^*\mu^*\nu^*+2\beta_1^*\beta_2\mu\nu+(|\beta_1|^2+|\beta_2|^2)(|\nu|^2+|\mu|^2)\right).\tag{S52}$$

One can reconcile these conditions, leading us to two different solutions: one which reproduces models in the rotating wave approximation (RWA), like the Jaynes-Cummings model, and another one which goes beyond the RWA and generates models like the generalized Rabi model.

A. RWA type models

The first possibility is to take the supercharge $\hat{\mathrm{Q}}_1$ in the following form

$$\hat{\mathrm{Q}}_1=\begin{pmatrix}0&0&\alpha&\gamma\hat{\mathrm{a}}\\0&0&\beta\hat{\mathrm{a}}^\dagger&\delta\\\alpha^*&\beta^*\hat{\mathrm{a}}&0&0\\\gamma^*\hat{\mathrm{a}}^\dagger&\delta^*&0&0\end{pmatrix}=\begin{pmatrix}0&\hat{\mathrm{q}}\\\hat{\mathrm{q}}^\dagger&0\end{pmatrix}.\tag{S53}$$

Its square reads

$$\hat{\mathrm{Q}}_1^2=\begin{pmatrix}|\alpha|^2+|\gamma|^2+|\gamma|^2\hat{\mathrm{a}}^\dagger\hat{\mathrm{a}}&g\,\hat{\mathrm{a}}&0&0\\g^*\hat{\mathrm{a}}^\dagger&|\beta|^2\hat{\mathrm{a}}^\dagger\hat{\mathrm{a}}+|\delta|^2&0&0\\0&0&|\alpha|^2+|\beta|^2+|\beta|^2\hat{\mathrm{a}}^\dagger\hat{\mathrm{a}}&\tilde{g}\,\hat{\mathrm{a}}\\0&0&\tilde{g}^*\hat{\mathrm{a}}^\dagger&|\gamma|^2\hat{\mathrm{a}}^\dagger\hat{\mathrm{a}}+|\delta|^2\end{pmatrix}=\begin{pmatrix}\hat{\mathrm{q}}\hat{\mathrm{q}}^\dagger&0\\0&\hat{\mathrm{q}}^\dagger\hat{\mathrm{q}}\end{pmatrix},\tag{S54}$$

where we set $g=\alpha\beta^*+\gamma\delta^*$, $\tilde{g}=\alpha^*\gamma+\beta^*\delta$. We request that

$$|\gamma|^2=\omega+\lambda,\qquad|\beta|^2=\omega-\lambda>0,\qquad|\alpha|^2+|\gamma|^2=\Delta_1+c_1,\qquad|\delta|^2=c_1-\Delta_1=c_2-\Delta_2>0,\tag{S55}$$

where $c_{1,2}$ are some constants that need to be determined. Further, we want

$$|\alpha|^2+|\beta|^2=\Delta_2+c_2,\tag{S56}$$

such that we can write $\hat{\mathbf{H}} = \hat{\mathrm{Q}}_1^2$, where the supersymmetric Hamiltonian is $\hat{\mathbf{H}} = \mathrm{diag}(\hat{\mathrm{H}}_+, \hat{\mathrm{H}}_-)$ with

$$\hat{\mathrm{H}}_+ = \hat{\mathrm{q}}\hat{\mathrm{q}}^\dagger = \omega \hat{\mathrm{a}}^\dagger \hat{\mathrm{a}} + \lambda\, \hat{\mathrm{a}}^\dagger \hat{\mathrm{a}}\, \hat{\sigma}_z + \Delta_1 \hat{\sigma}_z + g\, \hat{\mathrm{a}}\, \hat{\sigma}_+ + g^* \hat{\mathrm{a}}^\dagger \hat{\sigma}_- + c_1 \hat{1}, \tag{S57}$$

$$\hat{\mathrm{H}}_- = \hat{\mathrm{q}}^\dagger \hat{\mathrm{q}} = \omega \hat{\mathrm{a}}^\dagger \hat{\mathrm{a}} - \lambda\, \hat{\mathrm{a}}^\dagger \hat{\mathrm{a}}\, \hat{\sigma}_z + \Delta_2 \hat{\sigma}_z + \tilde{g}\, \hat{\mathrm{a}}\, \hat{\sigma}_+ + \tilde{g}^* \hat{\mathrm{a}}^\dagger \hat{\sigma}_- + c_2 \hat{1}. \tag{S58}$$

We note that the condition $|\alpha|^2 + |\beta|^2 = \Delta_2 + c_2$ can be rewritten as

$$\Delta_1 + c_1 - (\omega + \lambda) = \Delta_2 + c_2 - (\omega - \lambda). \tag{S59}$$

From this condition, together with the constrain imposed on $|\delta|^2$: $c_1 - \Delta_1 = c_2 - \Delta_2$, it follows that

$$\Delta_1 - \Delta_2 = \lambda. \tag{S60}$$

We focus on the case $\lambda = 0$ in order to compare our results with those from [S5]. $\lambda = 0$ implies that $\Delta_1 = \Delta_2 = \Delta$ and $c_1 = c_2 = c$. Further, we have

$$\beta = \gamma = \sqrt{\omega}, \qquad \alpha + \delta^* = \frac{g}{\sqrt{\omega}}, \qquad \alpha^* + \delta = \frac{\tilde{g}}{\sqrt{\omega}}. \tag{S61}$$

From $|\alpha|^2 + |\gamma|^2 = \Delta + c$, together with $|\delta|^2 = c - \Delta$, we obtain $|\alpha|^2 - |\delta|^2 = 2\Delta - \omega$. It is possible to assume that α and δ are real (i.e. $g = \tilde{g}$), meaning that $\alpha^2 - \delta^2 = 2\Delta - \omega$, together with $\alpha + \delta = g/\sqrt{\omega}$, implies that $\alpha - \delta = (2\Delta - \omega)\sqrt{\omega}/g$. We thus get

$$\alpha = \frac{1}{2}\left(\frac{g}{\sqrt{\omega}} + d\frac{\sqrt{\omega}}{g}\right), \qquad \delta = \frac{1}{2}\left(\frac{g}{\sqrt{\omega}} - d\frac{\sqrt{\omega}}{g}\right), \qquad c = \frac{1}{4}\left(\frac{g}{\sqrt{\omega}} - d\frac{\sqrt{\omega}}{g}\right)^2 + \Delta, \tag{S62}$$

where $d = 2\Delta - \omega$ is usually called detuning. The Jaynes-Cummings Hamiltonian, $\hat{\mathrm{H}}_{\mathrm{JC}} = \omega \hat{\mathrm{a}}^\dagger \hat{\mathrm{a}} + \Delta \hat{\sigma}_z + g(\hat{\mathrm{a}}\, \hat{\sigma}_+ + \hat{\mathrm{a}}^\dagger \hat{\sigma}_-)$, is hence part of the supersymmetric Hamiltonian $\hat{\mathbf{H}} = \hat{\mathrm{Q}}_1^2$, if $\lambda = 0$. Finally, we note that another possible solution would recover the results of [S5] in the limit of zero detuning, if we assume $\beta = \sqrt{\omega}$ and $\gamma = -i\sqrt{\omega}$.

B. Non-RWA type models

The second possibility is to take the supercharge $\hat{\mathrm{Q}}_1$ in the following form

$$\hat{\mathrm{Q}}_1 = \begin{pmatrix} 0 & 0 & \alpha & \gamma \hat{\mathrm{a}} \\ 0 & 0 & \beta \hat{\mathrm{a}} & \delta \\ \alpha^* & \beta^* \hat{\mathrm{a}}^\dagger & 0 & 0 \\ \gamma^* \hat{\mathrm{a}}^\dagger & \delta^* & 0 & 0 \end{pmatrix} = \begin{pmatrix} 0 & \hat{\mathrm{q}} \\ \hat{\mathrm{q}}^\dagger & 0 \end{pmatrix} \tag{S63}$$

and obtain for its square:

$$\hat{\mathrm{Q}}_1^2 = \begin{pmatrix} |\alpha|^2 + |\gamma|^2 + |\gamma|^2 \hat{\mathrm{a}}^\dagger \hat{\mathrm{a}} & \alpha\beta^* \hat{\mathrm{a}}^\dagger + \gamma\delta^* \hat{\mathrm{a}} & 0 & 0 \\ \alpha^* \beta\, \hat{\mathrm{a}} + \gamma^* \delta\, \hat{\mathrm{a}}^\dagger & |\beta|^2 \hat{\mathrm{a}}^\dagger \hat{\mathrm{a}} + |\beta|^2 + |\delta|^2 & 0 & 0 \\ 0 & 0 & |\alpha|^2 + |\beta|^2 \hat{\mathrm{a}}^\dagger \hat{\mathrm{a}} & \alpha^* \gamma\, \hat{\mathrm{a}} + \delta\beta^* \hat{\mathrm{a}}^\dagger \\ 0 & 0 & \alpha\gamma^* \hat{\mathrm{a}}^\dagger + \delta^* \beta\, \hat{\mathrm{a}} & |\gamma|^2 \hat{\mathrm{a}}^\dagger \hat{\mathrm{a}} + |\delta|^2 \end{pmatrix} = \begin{pmatrix} \hat{\mathrm{q}}\hat{\mathrm{q}}^\dagger & 0 \\ 0 & \hat{\mathrm{q}}^\dagger \hat{\mathrm{q}} \end{pmatrix}. \tag{S64}$$

Similar to the RWA case we set

$$\tilde{g}_1 = \gamma\delta^*, \qquad \tilde{g}_2 = \alpha\beta^*, \qquad g_1 = \alpha^* \gamma, \qquad g_2 = \beta^* \delta, \tag{S65}$$

and we request that

$$|\gamma|^2 = \omega + \lambda, \qquad |\beta|^2 = \omega - \lambda, \tag{S66}$$

such that $\omega > \lambda$. Further, we require

$$|\alpha|^2 + |\gamma|^2 = c_1 + \frac{\Delta}{2}, \qquad |\beta|^2 + |\delta|^2 = c_1 - \frac{\Delta}{2}, \qquad |\alpha|^2 = c_2 + \frac{\Delta}{2} - \lambda, \qquad |\delta|^2 = c_2 - \frac{\Delta}{2} + \lambda, \tag{S67}$$

where c_1, c_2 are constants. These conditions allow us to write $\hat{\mathbf{H}} = \hat{\mathrm{Q}}_1^2$, where the supersymmetric Hamiltonian, $\hat{\mathbf{H}} = \mathrm{diag}(\hat{\mathrm{H}}_+, \hat{\mathrm{H}}_-)$, is given by

$$\hat{\mathrm{H}}_+ = \hat{\mathrm{q}}\hat{\mathrm{q}}^\dagger = \omega\hat{\mathrm{a}}^\dagger\hat{\mathrm{a}} + \frac{\Delta}{2}\hat{\sigma}_z + \lambda\hat{\mathrm{a}}^\dagger\hat{\mathrm{a}}\,\hat{\sigma}_z + \tilde{g}_1^*\hat{\mathrm{a}}^\dagger\hat{\sigma}_- + \tilde{g}_1\hat{\mathrm{a}}\hat{\sigma}_+ + \tilde{g}_2\hat{\mathrm{a}}^\dagger\hat{\sigma}_+ + \tilde{g}_2^*\hat{\mathrm{a}}\hat{\sigma}_- + c_1\hat{1}, \tag{S68}$$

$$\hat{\mathrm{H}}_- = \hat{\mathrm{q}}^\dagger\hat{\mathrm{q}} = \omega\hat{\mathrm{a}}^\dagger\hat{\mathrm{a}} + (\frac{\Delta}{2} - \lambda)\hat{\sigma}_z - \lambda\hat{\mathrm{a}}^\dagger\hat{\mathrm{a}}\,\hat{\sigma}_z + g_1^*\hat{\mathrm{a}}^\dagger\hat{\sigma}_- + g_1\hat{\mathrm{a}}\hat{\sigma}_+ + g_2\hat{\mathrm{a}}^\dagger\hat{\sigma}_+ + g_2^*\hat{\mathrm{a}}\hat{\sigma}_- + c_2\hat{1}. \tag{S69}$$

The conditions (S67) imply that

$$c_2 = c_1 - \omega, \qquad c_1 = \frac{1}{2}(|\alpha|^2 + |\delta|^2) + \omega, \qquad \frac{\Delta}{2} = \frac{1}{2}(|\alpha|^2 - |\delta|^2) + \lambda, \tag{S70}$$

and from the conditions (S65) and (S66) we get

$$\alpha = \frac{\tilde{g}_2}{\sqrt{\omega - \lambda}}, \qquad \alpha^* = \frac{g_1}{\sqrt{\omega + \lambda}}, \qquad \delta = \frac{g_2}{\sqrt{\omega - \lambda}}, \qquad \delta^* = \frac{\tilde{g}_1}{\sqrt{\omega + \lambda}}. \tag{S71}$$

Let us assume that α and δ are real, then we have $\tilde{g}_2/\sqrt{\omega - \lambda} = g_1/\sqrt{\omega + \lambda}$ and $g_2/\sqrt{\omega - \lambda} = \tilde{g}_1/\sqrt{\omega + \lambda}$, and we find

$$c_1 = \frac{1}{2}\left(\frac{\omega(g_1^2 + g_2^2) - \lambda(g_1^2 - g_2^2)}{\omega^2 - \lambda^2}\right) + \omega \tag{S72}$$

as well as the condition

$$2(\frac{\Delta}{2} - \lambda)(\omega + \lambda)(\omega - \lambda) = (\omega - \lambda)g_1^2 - (\omega + \lambda)g_2^2, \tag{S73}$$

which needs to be satisfied if we want to have $\hat{\mathbf{H}} = \hat{\mathrm{Q}}_1^2$.

In the case of $\lambda = 0$, we have

$$\hat{\mathrm{H}}_+ = \hat{\mathrm{q}}\hat{\mathrm{q}}^\dagger = \omega\hat{\mathrm{a}}^\dagger\hat{\mathrm{a}} + \frac{\Delta}{2}\hat{\sigma}_z + g_2\hat{\mathrm{a}}^\dagger\hat{\sigma}_- + g_2\hat{\mathrm{a}}\hat{\sigma}_+ + g_1\hat{\mathrm{a}}^\dagger\hat{\sigma}_+ + g_1\hat{\mathrm{a}}\hat{\sigma}_- + c_1\hat{1}, \tag{S74}$$

$$\hat{\mathrm{H}}_- = \hat{\mathrm{q}}^\dagger\hat{\mathrm{q}} = \omega\hat{\mathrm{a}}^\dagger\hat{\mathrm{a}} + \frac{\Delta}{2}\hat{\sigma}_z + g_1\hat{\mathrm{a}}^\dagger\hat{\sigma}_- + g_1\hat{\mathrm{a}}\hat{\sigma}_+ + g_2\hat{\mathrm{a}}^\dagger\hat{\sigma}_+ + g_2\hat{\mathrm{a}}\hat{\sigma}_- + (c_1 - \omega)\hat{1}, \tag{S75}$$

with $c_1 = \frac{1}{2}\frac{g_1^2+g_2^2}{\omega} + \omega$. If the condition $\Delta\omega = g_1^2 - g_2^2$ is satisfied, we can write $\hat{\mathbf{H}} = \hat{\mathrm{Q}}_1^2$, with

$$\hat{\mathrm{Q}}_1 = \begin{pmatrix} 0 & 0 & \frac{g_1}{\sqrt{\omega}} & \omega\hat{\mathrm{a}} \\ 0 & 0 & \omega\hat{\mathrm{a}} & \frac{g_2}{\sqrt{\omega}} \\ \frac{g_1}{\sqrt{\omega}} & \omega\hat{\mathrm{a}}^\dagger & 0 & 0 \\ \omega\hat{\mathrm{a}}^\dagger & \frac{g_2}{\sqrt{\omega}} & 0 & 0 \end{pmatrix} = \begin{pmatrix} 0 & \hat{\mathrm{q}} \\ \hat{\mathrm{q}}^\dagger & 0 \end{pmatrix}. \tag{S76}$$

The generalized Rabi model is therefore part of the supersymmetric system through $\hat{\mathrm{H}}_- = \hat{\mathrm{H}}_{\mathrm{gR}} + (c_1 - \omega)\hat{1}$. The condition $\Delta\omega = g_1^2 - g_2^2$ shows that the Rabi model ($g_1 = g_2 = g$) can become supersymmetric only in the special case of $\Delta = 0$.

Finally, we remark that we could have added other non-linearities to the Hamiltonian $\hat{\mathbf{H}}$, in particular the terms $\hat{\sigma}_z\hat{\mathrm{a}}^2 + \hat{\sigma}_z(\hat{\mathrm{a}}^\dagger)^2$. These terms could come from the quantization of the A^2 term (square of the vector potential, which appears beyond the dipole approximation) in the Hamiltonian, $\hat{\mathrm{H}}_+$ or $\hat{\mathrm{H}}_-$, describing the actual physical system, as well as from other radiative corrections.

C. Zero-mode eigenfunctions

In this subsection, we determine the zero-mode eigenstates $|\Psi_0\rangle$. These states obey $\hat{\mathbf{H}}|\Psi_0\rangle = 0$, as they are the eigenstates with zero eigenvalues of the supersymmetric Hamiltonian $\hat{\mathbf{H}} = \hat{\mathrm{Q}}_1^2$. The supersymmetric Hamiltonian $\hat{\mathbf{H}} = \mathrm{diag}(\hat{\mathrm{H}}_+, \hat{\mathrm{H}}_-)$ consists of two different Hamiltonians $\hat{\mathrm{H}}_+$, $\hat{\mathrm{H}}_-$ with positive and negative Witten parity, respectively,

which have the same eigenstates apart from the zero-modes. We can establish the zero-modes of $\hat{\mathrm{H}}_+$ and $\hat{\mathrm{H}}_-$ separately since we have

$$\hat{\mathbf{H}}|\Psi_0\rangle = \begin{pmatrix} \hat{\mathrm{H}}_+ & 0 \\ 0 & \hat{\mathrm{H}}_- \end{pmatrix} \begin{pmatrix} |\Psi_0^+\rangle \\ |\Psi_0^-\rangle \end{pmatrix} = \begin{pmatrix} \hat{\mathrm{q}}\hat{\mathrm{q}}^\dagger & 0 \\ 0 & \hat{\mathrm{q}}^\dagger\hat{\mathrm{q}} \end{pmatrix} \begin{pmatrix} |\Psi_0^+\rangle \\ |\Psi_0^-\rangle \end{pmatrix} = 0. \tag{S77}$$

This yields the two separate equations

$$\hat{\mathrm{q}}^\dagger\,|\Psi_0^+\rangle = 0, \qquad \hat{\mathrm{q}}\,|\Psi_0^-\rangle = 0, \tag{S78}$$

for the zero-modes $|\Psi_0^+\rangle$, $|\Psi_0^-\rangle$ of $\hat{\mathrm{H}}_+$, $\hat{\mathrm{H}}_-$, respectively.

Let us focus on the case where the generalized Rabi model is part of the supersymmetric system. In this case we have $\hat{\mathbf{H}} = \mathrm{diag}(\hat{\mathrm{H}}_+, \hat{\mathrm{H}}_-)$ with

$$\hat{\mathrm{H}}_+ = \omega\hat{\mathrm{a}}^\dagger\hat{\mathrm{a}} + \frac{\Delta}{2}\hat{\sigma}_z + g_2\hat{\mathrm{a}}^\dagger\hat{\sigma}_- + g_2\hat{\mathrm{a}}\hat{\sigma}_+ + g_1\hat{\mathrm{a}}^\dagger\hat{\sigma}_+ + g_1\hat{\mathrm{a}}\hat{\sigma}_- + (c+\omega)\hat{1}, \tag{S79}$$

$$\hat{\mathrm{H}}_- = \omega\hat{\mathrm{a}}^\dagger\hat{\mathrm{a}} + \frac{\Delta}{2}\hat{\sigma}_z + g_1\hat{\mathrm{a}}^\dagger\hat{\sigma}_- + g_1\hat{\mathrm{a}}\hat{\sigma}_+ + g_2\hat{\mathrm{a}}^\dagger\hat{\sigma}_+ + g_2\hat{\mathrm{a}}\hat{\sigma}_- + (c-\omega)\hat{1} = \hat{\mathrm{H}}_{\mathrm{gR}} + c\hat{1}, \tag{S80}$$

where $c = \frac{1}{2}\frac{g_1^2+g_2^2}{\omega}$. If the parameters satisfy the condition $\Delta\omega = g_1^2 - g_2^2$, we can write $\hat{\mathbf{H}} = \hat{\mathrm{Q}}_1^2$, with the supercharge given by

$$\hat{\mathrm{Q}}_1 = \begin{pmatrix} 0 & 0 & \frac{g_1}{\sqrt{\omega}} & \omega\hat{\mathrm{a}} \\ 0 & 0 & \omega\hat{\mathrm{a}} & \frac{g_2}{\sqrt{\omega}} \\ \frac{g_1}{\sqrt{\omega}} & \omega\hat{\mathrm{a}}^\dagger & 0 & 0 \\ \omega\hat{\mathrm{a}}^\dagger & \frac{g_2}{\sqrt{\omega}} & 0 & 0 \end{pmatrix} = \begin{pmatrix} 0 & \hat{\mathrm{q}} \\ \hat{\mathrm{q}}^\dagger & 0 \end{pmatrix}, \tag{S81}$$

as we derived in the previous subsection S2 B. The zero-modes of $\hat{\mathrm{H}}_-$ are then determined by $\hat{\mathrm{q}}\,|\Psi_0^-\rangle = 0$, which yields following the equations

$$\frac{g_1}{\sqrt{\omega}}|\chi_0^-\rangle + \sqrt{\omega}\hat{\mathrm{a}}|\phi_0^-\rangle = 0, \tag{S82}$$

$$\sqrt{\omega}\hat{\mathrm{a}}|\chi_0^-\rangle + \frac{g_2}{\sqrt{\omega}}|\phi_0^-\rangle = 0, \tag{S83}$$

where $|\Psi_0^-\rangle = (|\chi_0^-\rangle, |\phi_0^-\rangle)^T$. Expanding the possible solutions $|\chi_0^-\rangle$ and $|\phi_0^-\rangle$ as a series expansion in the Fock basis

$$|\chi_0^-\rangle = \sum_{n=0}^{\infty} c(n)|n\rangle, \qquad |\phi_0^-\rangle = \sum_{n=0}^{\infty} d(n)|n\rangle, \tag{S84}$$

leads to the following equations for $c(n)$ and $d(n)$

$$\frac{g_1}{\sqrt{\omega}}\sum_{n=0}^{\infty} c(n)|n\rangle + \sqrt{\omega}\sum_{n=0}^{\infty} d(n)\sqrt{n}|n-1\rangle = 0, \tag{S85}$$

$$\sqrt{\omega}\sum_{n=0}^{\infty} c(n)\sqrt{n}|n-1\rangle + \frac{g_2}{\sqrt{\omega}}\sum_{n=0}^{\infty} d(n)|n\rangle = 0. \tag{S86}$$

We note that the physically allowed solutions of $c(n)$ and $d(n)$ must be absolute convergent sums, i.e., $\sum_n^\infty |c(n)|^2 < \infty$ and $\sum_n^\infty |d(n)|^2 < \infty$. By equating the coefficients at the same state $|n\rangle$, we obtain the system

$$g_1\,c(n) + \omega\,\sqrt{n+1}\,d(n+1) = 0, \tag{S87}$$

$$\omega\,\sqrt{n+1}\,c(n+1) + g_2\,d(n) = 0, \tag{S88}$$

from which we get

$$d(n) = \frac{\left(\pm\frac{\sqrt{g_1 g_2}}{\omega}\right)^n}{\sqrt{n!}}. \tag{S89}$$

The normalization condition $\langle\Psi_0|\Psi_0\rangle = 1$, together with (S87), yield the two zero-modes

$$|\Psi_0^-\rangle = \frac{1}{\sqrt{2}}\left(|\nu\rangle|\uparrow\rangle \pm |-\nu\rangle|\downarrow\rangle\right), \tag{S90}$$

where

$$|\nu\rangle = e^{-\frac{|\nu|^2}{2}}\sum_{n=0}^{\infty}\frac{\nu^n}{\sqrt{n!}}|n\rangle = e^{-\frac{|\nu|^2}{2}}e^{\nu\hat{\mathrm{a}}^\dagger}|0\rangle \tag{S91}$$

is a coherent state with $\nu = \sqrt{g_1 g_2}/\omega$, and where $|\uparrow\rangle$, $|\downarrow\rangle$ are the eigenstates of $\hat{\sigma}_z$.

A similar reasoning shows that there are no absolute convergent solutions to $\hat{\mathrm{q}}^\dagger\,|\Psi_0^+\rangle = 0$ and therefore $\hat{\mathrm{H}}_+$ has no zero-modes. We have an unbroken SUSY with the two zero modes,

$$|\Psi_0\rangle = \frac{1}{\sqrt{2}}\begin{pmatrix} 0 \\ 0 \\ |\nu\rangle \\ \pm|-\nu\rangle \end{pmatrix}. \tag{S92}$$

S3. DISSIPATION: DEGENERATE ZERO EIGENVALUES OF A LIOUVILLIAN

Let us consider the following master equation for a density matrix

$$i\frac{d}{dt}|\rho(t)\rangle\rangle = L|\rho(t)\rangle\rangle. \tag{S93}$$

We use Dirac-like notations to indicate that, in the Liouvillian space, a density matrix is represented by a vector. The Liouvillian L is a non-Hermitian operator, therefore its eigenvalues are complex-valued. Moreover, in a physically meaningful model their imaginary parts must be smaller than zero (causality principle – no exponentially growing solutions).

A. Topological arguments

We focus here on the case of zero dephasing, $\Phi_j = 0$. First, we note that the stationary density matrix $|\rho_{st}\rangle\rangle$ is annihilated by $\hat{\mathrm{Q}}$, $\hat{\mathrm{Q}}|\rho_{st}\rangle\rangle = 0$, on the SUSY line $g_1^2 - g_2^2 = \Delta\omega$. The dimension of the ground state manifold is directly related to the dimension of the *cohomology* space $\mathcal{H}_Q$ of the SUSY $\hat{\mathrm{Q}}$-operator, $\dim(\mathcal{H}_Q) = \dim(\ker(\hat{\mathrm{Q}})/\mathrm{im}(\hat{\mathrm{Q}}))$, where $\ker(\hat{\mathrm{Q}})$ is the space of solutions $\hat{\mathrm{Q}}|\chi\rangle = 0$, while $\mathrm{im}(\hat{\mathrm{Q}})$ is the space of all states which can be written as $\hat{\mathrm{Q}}|\psi\rangle$ for some $|\psi\rangle$. This dimension in our case is equal to 2. It was shown by Witten in [S6] that $\dim(\mathcal{H}_Q)$ is unchanged by one-parametric family of non-unitary transformations. In our context these non-unitary conjugations are generated by the dissipative evolution operator, $\exp(-iLt)$, where L is the Liouvillian. Therefore, we have $\dim(|\rho_{st}\rangle\rangle) = (\dim(\mathcal{H}_Q))^2$. The stationary state is thus insensitive to the details of L. In addition, the Witten index, being a topological invariant according to the Atiah-Singer index theorem, is also invariant under a large class of deformations. In our case, $W_{\mathrm{ind}} = \dim(\mathcal{H}_Q) = 2$, which justifies why $\hat{\rho}_{st}$ is a two by two matrix. This, combined with the unit trace condition and the hermiticity, gives an equivalence to the SU(2) group.

B. General theory of degenerate Liouvillian

Suppose that we have a peculiar situation, where there are more than one zero eigenvalue of L (more precisely, $-i0^+$). We look for the corresponding left and right eigenvectors of L

$$\langle\langle\bar{\rho}^{(i)}|L = 0, \tag{S94}$$

$$L|\rho^{(i)}\rangle\rangle = 0, \tag{S95}$$

where $i = 1, \ldots, r$, and r is the degeneracy. Note that for a general non-Hermitian operator, left and right eigenvalues are not related to each other by any operation (conjugation, transposition, etc.), and thus they are independent of each other, besides the condition

$$\langle\langle\bar{\rho}^{(i)}|\rho^{(j)}\rangle\rangle = \delta_{ij}, \qquad i,j = 1,\ldots,r. \tag{S96}$$

As a consequence, these two eigenvalue problems have to be solved separately. This is explicitly reflected by an additional bar symbol on the left eigenvector. The projector onto the zero eigenspace is, then given by

$$P_0 = \sum_{i=1}^{r} |\rho^{(i)}\rangle\rangle\langle\langle\bar{\rho}^{(i)}|. \tag{S97}$$

Let us now write a solution for time dynamics [Eq. (S93)]

$$|\rho(t)\rangle\rangle = e^{-iLt}|\rho(0)\rangle\rangle. \tag{S98}$$

Representing the Liouvillian

$$L = \sum_{s=r+1}^{n^2} \lambda_s P_s \tag{S99}$$

by a sum over all non-zero eigenvalues times the corresponding projectors (where n is the dimension of the Hilbert space of the system), we find

$$|\rho(t)\rangle\rangle = \left[1 + \sum_s P_s\left(e^{-i\lambda_s t} - 1\right)\right]|\rho(0)\rangle\rangle = \left[P_0 + \sum_s P_s e^{-i\lambda_s t}\right]|\rho(0)\rangle\rangle, \tag{S100}$$

where we used $P_0 + \sum_s P_s = 1$.

In the long time limit we obtain the stationary density matrix

$$|\rho(t\to\infty)\rangle\rangle \equiv |\rho_{st}\rangle\rangle = P_0|\rho(0)\rangle\rangle = \sum_i |\rho^{(i)}\rangle\rangle\langle\langle\bar\rho^{(i)}|\rho(0)\rangle\rangle. \tag{S101}$$

Let us now consider the quantities $I_i = \langle\langle\bar\rho^{(i)}|\rho(0)\rangle\rangle$. In this form, they appear to depend on the initial conditions (however, in absence of degeneracy this dependence is gone, see below). Acting on (S93) with P_0, we find

$$\sum_i |\rho^{(i)}\rangle\rangle \frac{d}{dt}\langle\langle\bar\rho^{(i)}|\rho(t)\rangle\rangle = 0. \tag{S102}$$

Since all $|\rho^{(i)}\rangle\rangle$ are linearly independent, we find that $I_i(t) = \langle\langle\bar\rho^{(i)}|\rho(t)\rangle\rangle$ are conserved quantities, $I_i(t) = I_i$.

The values of (most of) I_i are fixed by the initial conditions. But there is one conserved quantity, namely $\mathrm{Tr}[\hat\rho(t)] = 1$, which is independent of the initial conditions. This means that the basis in the degenerate zero subspace can always be chosen in such a way that one left eigenvector appears to be $\langle\langle\bar\rho^{(tr)}| = (1,\ldots,1,0,\ldots,0)$, where entries 1 appear in positions of diagonal density matrix elements (n times), and entries 0 appear in positions of non-diagonal elements ($n^2 - n$ times). Thus, we have $\langle\langle\bar\rho^{(tr)}|\rho(t)\rangle\rangle = \mathrm{Tr}[\hat\rho(t)] = 1$.

In absence of degeneracy, $\langle\langle\bar\rho^{(tr)}|$ is the only (left) eigenvector, and therefore the stationary density matrix reads

$$|\rho_{st}\rangle\rangle = |\rho^{(tr)}\rangle\rangle, \tag{S103}$$

i.e., it coincides with the corresponding right eigenvector. Thereby, all information about the initial conditions is lost, as $|\rho^{(tr)}\rangle\rangle$ depends only on L.

The knowledge of $\hat\rho_{st}$ allows us to find expectation values of observables in the stationary regime.

C. Relaxation in the generalized Rabi model

Define

$$\hat{\bar\rho}^{(i'j')} = \sum_{k,k'} \bar\rho^{(i'j')}_{kk'}|k\rangle\langle k'|, \tag{S104}$$

which is equivalent to

$$|\bar\rho^{(i'j')}\rangle\rangle = \sum_{k,k'} \bar\rho^{(i'j')}_{kk'}|kk'\rangle\rangle, \qquad \langle\langle\bar\rho^{(i'j')}| = \sum_{k,k'} \bar\rho^{(i'j')*}_{kk'}\langle\langle kk'| = \sum_{k,k'} (\bar\rho^{(i'j')\dagger})_{k'k}\langle\langle kk'|, \tag{S105}$$

where

$$|k\rangle\langle k'| = |kk'\rangle\rangle = \langle\langle k'k| \tag{S106}$$

and i', j' count the degeneracy. It is apparent that $\langle\langle ll'|kk'\rangle\rangle = \mathrm{Tr}[|l'\rangle\langle l|k\rangle\langle k'|] = \delta_{lk}\delta_{l'k'}$, and therefore

$$\langle\langle\bar\rho^{(i'j')}|\rho^{(ij)}\rangle\rangle = \sum_{l,l',k,k'} (\bar\rho^{(i'j')\dagger})_{l'l}\rho^{(ij)}_{kk'}\langle\langle ll'|kk'\rangle\rangle = \mathrm{Tr}[\hat{\bar\rho}^{(i'j')\dagger}\hat\rho^{(ij)}]. \tag{S107}$$

First, we determine the subspace of stationary density matrices solving the equation

$$0 = -i[\hat{\mathrm{H}}, \hat\rho^{(ij)}] + \mathcal{D}\left[\sum_k \Phi_k|k\rangle\langle k|\right]\hat\rho^{(ij)} + \sum_{k'>k}\mathcal{D}\left[\hat{\mathrm{O}}^{(kk')}\right]\hat\rho^{(ij)}, \tag{S108}$$

where $\hat{\mathrm{O}}^{(kk')} = \sqrt{\Gamma^{(kk')}}|k\rangle\langle k'|$, and

$$\mathcal{D}[\hat{\mathrm{O}}]\hat{\rho} \;=\; \hat{\mathrm{O}}\,\hat{\rho}\,\hat{\mathrm{O}}^\dagger - \frac{1}{2}\hat{\rho}\,\hat{\mathrm{O}}^\dagger\,\hat{\mathrm{O}} - \frac{1}{2}\hat{\mathrm{O}}^\dagger\,\hat{\mathrm{O}}\,\hat{\rho}, \tag{S109}$$

$$\hat{\mathrm{H}} \;=\; \sum_k \varepsilon_k |k\rangle\langle k|. \tag{S110}$$

Note that $\Gamma^{(kk')} \neq 0$ only for $k' > k$.

Suppose now that $\varepsilon_1 = \varepsilon_2$, and $\Gamma^{(12)} = 0$. We find that: 1) There are four solutions $\hat{\rho}^{(ij)} = |i\rangle\langle j|$, $i, j = 1, 2$, to the equation (S108) for $\Phi_1 = \Phi_2$ and 2) there are two solutions $\hat{\rho}^{(11)} = |1\rangle\langle 1|$ and $\hat{\rho}^{(22)} = |2\rangle\langle 2|$ for $\Phi_1 \neq \Phi_2$.

Next, we establish the conserved quantities $\hat{\bar{\rho}}^{(ij)} = \sum_{kk'} \bar{\rho}^{(ij)}_{kk'} |k\rangle\langle k'|$ by solving the equations

$$\langle\!\langle \bar{\rho}^{(i'j')} | \rho^{(ij)} \rangle\!\rangle = \delta_{ii'}\delta_{jj'} \tag{S111}$$

and

$$0 = i[\hat{\mathrm{H}}, \hat{\bar{\rho}}^{(ij)}] + \mathcal{D}^\dagger \left[\sum_k \Phi_k |k\rangle\langle k| \right] \hat{\bar{\rho}}^{(ij)} + \sum_{k'>k} \mathcal{D}^\dagger \left[\hat{\mathrm{O}}^{(kk')} \right] \hat{\bar{\rho}}^{(ij)}, \tag{S112}$$

where

$$\mathcal{D}^\dagger[\hat{\mathrm{O}}]\hat{\rho} = \hat{\mathrm{O}}^\dagger\,\hat{\rho}\,\hat{\mathrm{O}} - \frac{1}{2}\hat{\rho}\,\hat{\mathrm{O}}^\dagger\,\hat{\mathrm{O}} - \frac{1}{2}\hat{\mathrm{O}}^\dagger\,\hat{\mathrm{O}}\,\hat{\rho}. \tag{S113}$$

The condition (S111) implies that

$$\bar{\rho}^{(ij)}_{kk'} = \delta_{ik}\delta_{jk'}, \qquad i, j = 1, 2; \qquad k, k' = 1, 2. \tag{S114}$$

The other components k, k' of $\hat{\bar{\rho}}^{(ij)}$ should be found from the equation (S112).

It is easy to check that $\hat{\bar{\rho}}^{(12)} = |1\rangle\langle 2|$ and $\hat{\bar{\rho}}^{(21)} = |2\rangle\langle 1|$ for $\Phi_1 = \Phi_2$, and for $\Phi_1 \neq \Phi_2$ these states are of no interest. Therefore, we can concentrate on the diagonal components $\hat{\bar{\rho}}^{(ii)} \equiv \hat{\bar{\rho}}^{(i)}$, $i = 1, 2$, such that $\bar{\rho}^{(1)}_{11} = \bar{\rho}^{(2)}_{22} = 1$ and $\bar{\rho}^{(1)}_{22} = \bar{\rho}^{(2)}_{11} = 0$. We rewrite the equation (S112) for $\bar{\rho}^{(i)}_{kk'}$ as

$$\begin{aligned} 0 \;=\; & i \sum_{k=3}^{\infty} \sum_{k'=3}^{\infty} (\varepsilon_k - \varepsilon_{k'}) \bar{\rho}^{(i)}_{kk'} |k\rangle\langle k'| \\ & + \sum_{k=3}^{\infty} \Gamma^{(ik)} |k\rangle\langle k| - \sum_{k=3}^{\infty} \sum_{k'=3}^{\infty} \frac{\Gamma^{(1k)} + \Gamma^{(1k')} + \Gamma^{(2k)} + \Gamma^{(2k')}}{2} \bar{\rho}^{(i)}_{kk'} |k\rangle\langle k'| \\ & + \sum_{k=4}^{\infty} \left(\sum_{l=3}^{k-1} \Gamma^{(lk)} \bar{\rho}^{(i)}_{ll} \right) |k\rangle\langle k| - \sum_{k=4}^{\infty} \left(\sum_{l=3}^{k-1} \frac{\Gamma^{(lk)}}{2} \right) \sum_{k'=1}^{\infty} \bar{\rho}^{(i)}_{kk'} |k\rangle\langle k'| - \sum_{k'=4}^{\infty} \left(\sum_{l=3}^{k'-1} \frac{\Gamma^{(lk')}}{2} \right) \sum_{k=1}^{\infty} \bar{\rho}^{(i)}_{kk'} |k\rangle\langle k'|. \end{aligned} \tag{S115}$$

Note that the term with Φ_k drops out. We immediately see that $\bar{\rho}^{(i)}_{kk'} = 0$ for $k \neq k'$ because the corresponding equation is homogeneous. The equation for the diagonal components $\bar{\rho}^{(i)}_{kk} \equiv \bar{\rho}^{(i)}_k$ simplifies to

$$\begin{aligned} 0 \;=\; & \sum_{k=3}^{\infty} \Gamma^{(ik)} |k\rangle\langle k| - \sum_{k=3}^{\infty} (\Gamma^{(1k)} + \Gamma^{(2k)}) \bar{\rho}^{(i)}_k |k\rangle\langle k| \\ & + \sum_{k=4}^{\infty} \left(\sum_{l=3}^{k-1} \Gamma^{(lk)} \bar{\rho}^{(i)}_l \right) |k\rangle\langle k| - \sum_{k=4}^{\infty} \left(\sum_{l=3}^{k-1} \Gamma^{(lk)} \right) \bar{\rho}^{(i)}_k |k\rangle\langle k| \\ =\; & \Gamma^{(i3)} |3\rangle\langle 3| - (\Gamma^{(13)} + \Gamma^{(23)}) \bar{\rho}^{(i)}_3 |3\rangle\langle 3| \\ & + \sum_{k=4}^{\infty} \left(\Gamma^{(ik)} + \sum_{l=3}^{k-1} \Gamma^{(lk)} \bar{\rho}^{(i)}_l \right) |k\rangle\langle k| - \sum_{k=4}^{\infty} \left(\sum_{l=1}^{k-1} \Gamma^{(lk)} \right) \bar{\rho}^{(i)}_k |k\rangle\langle k|, \end{aligned} \tag{S116}$$

from which we conclude

$$\bar{\rho}_3^{(i)} = \frac{\Gamma^{(i3)}}{\Gamma^{(13)} + \Gamma^{(23)}}, \tag{S117}$$

$$\bar{\rho}_k^{(i)} = \frac{\Gamma^{(ik)} + \sum_{l=3}^{k-1} \Gamma^{(lk)} \bar{\rho}_l^{(i)}}{\sum_{l=1}^{k-1} \Gamma^{(lk)}}. \tag{S118}$$

The latter expression is a recurrence relation, which allows us to evaluate $\bar{\rho}_k^{(i)}$, if all $\bar{\rho}_{l<k}^{(i)}$ are known (recall that $\rho_1^{(1)} = \rho_2^{(2)} = 1$ and $\rho_2^{(1)} = \rho_1^{(2)} = 0$).

We point out that $\hat{\hat{\rho}}^{(1)} + \hat{\hat{\rho}}^{(2)} = \hat{\hat{\rho}}^{(tr)} = \hat{1}$: for $k = 1, 2, 3$ this is obvious, while for $k \geq 4$ we obtain

$$\bar{\rho}_k^{(tr)} = \frac{\Gamma^{(1k)} + \Gamma^{(2k)} + \sum_{l=3}^{k-1} \Gamma^{(lk)} \bar{\rho}_l^{(tr)}}{\sum_{l=1}^{k-1} \Gamma^{(lk)}}. \tag{S119}$$

If $\bar{\rho}_3^{(tr)} = 1$, then $\bar{\rho}_4^{(tr)} = 1$, and so forth, which leads to $\bar{\rho}_k^{(tr)} = 1$ for all k.

The matrix $\hat{\hat{\rho}}^{(tr)}$ is conjugated to the stationary matrix $\hat{\rho}_{st} = \frac{1}{2}(|1\rangle\langle 1| + |2\rangle\langle 2|)$. Another pair of conjugated matrices is $\hat{\hat{\rho}}^{(diff)} = \hat{\hat{\rho}}^{(1)} - \hat{\hat{\rho}}^{(2)}$ and $\hat{\rho}_{diff} = \frac{1}{2}(|1\rangle\langle 1| - |2\rangle\langle 2|)$.

According to (S101) we find

$$\begin{aligned} \hat{\rho}(t \to \infty) &= \frac{1}{2}(|1\rangle\langle 1| + |2\rangle\langle 2|) + \frac{1}{2}(|1\rangle\langle 1| - |2\rangle\langle 2|)\mathrm{Tr}\left[(\hat{\hat{\rho}}^{(1)} - \hat{\hat{\rho}}^{(2)})\hat{\rho}(0)\right] \\ &+ |1\rangle\langle 1|\hat{\rho}(0)|2\rangle\langle 2| + |2\rangle\langle 2|\hat{\rho}(0)|1\rangle\langle 1|, \end{aligned} \tag{S120}$$

where the second line needs to be added only for the case $\Phi_1 = \Phi_2$ (e.g., when both of them are zero).

[S1] Baksic, A. & Ciuti, C. Controlling discrete and continuous symmetries in "superradiant" phase transitions with circuit QED systems. *Phys. Rev. Lett.* **112**, 173601 (2014).

[S2] Cho, J., Angelakis, D. G. & Bose, S. Simulation of high-spin Heisenberg models in coupled cavities. *Phys. Rev. A* **78**, 062338 (2008).

[S3] Magnus, W. On the exponential solution of differential equations for a linear operator. *Commun. Pure Appl. Math.* **7**, 649-673 (1954).

[S4] Dimer, F., Estienne, B., Parkins, A. S. & Carmichael, H. J. Proposed realization of the Dicke-model quantum phase transition in an optical cavity QED system. *Phys. Rev. A* **75**, 013804 (2007).

[S5] Andreev, V. A. & Lerner, P. B. Supersymmetry in the Jaynes-Cummings model. *Phys. Lett. A* **134**, 507-511 (1989).

[S6] Witten, E. Constraints on supersymmetry breaking. *Nucl. Phys. B* **202**, 253 (1982).
